# Chemical Species Ontology for Data Integration and Knowledge Discovery

**DOI:** 10.1021/acs.jcim.3c00820

**Published:** 2023-10-26

**Authors:** Laura Pascazio, Simon Rihm, Ali Naseri, Sebastian Mosbach, Jethro Akroyd, Markus Kraft

**Affiliations:** † CARES, Cambridge Centre for Advanced Research and Education in Singapore, 1 Create Way, CREATE Tower, #05-05, Singapore 138602, Singapore; ‡ Department of Chemical Engineering and Biotechnology, University of Cambridge, Philippa Fawcett Drive, Cambridge CB3 0AS, U.K.; § CMCL Innovations, Sheraton House, Castle Park, Cambridge CB3 0AX, U.K.; ∥ School of Chemical and Biomedical Engineering, Nanyang Technological University, 62 Nanyang Drive, Singapore 637459, Singapore; ⊥ The Alan Turing Institute, 96 Euston Rd., London NW1 2DB, U.K.

## Abstract

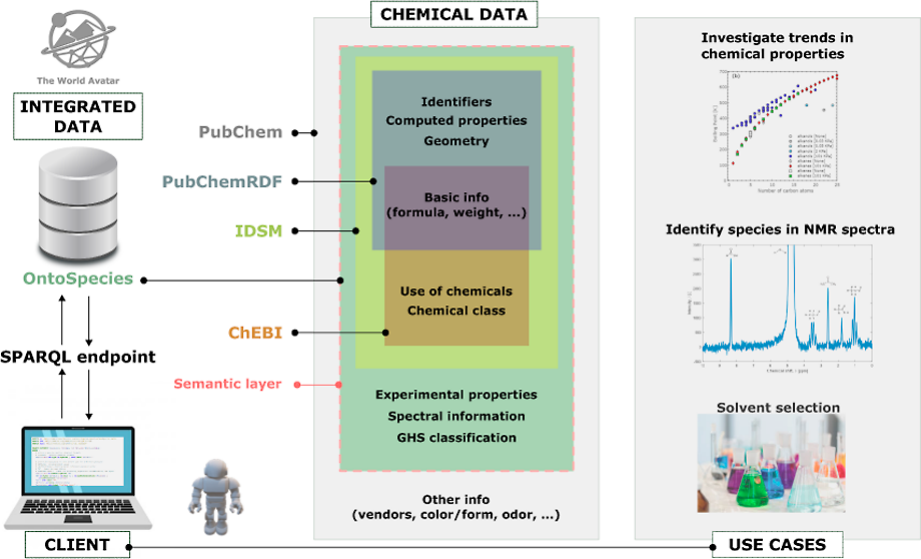

Web ontologies are important tools in modern scientific research because they provide a standardized way to represent and manage web-scale amounts of complex data. In chemistry, a semantic database for chemical species is indispensable for its ability to interrelate and infer relationships, enabling a more precise analysis and prediction of chemical behavior. This paper presents OntoSpecies, a web ontology designed to represent chemical species and their properties. The ontology serves as a core component of The World Avatar knowledge graph chemistry domain and includes a wide range of identifiers, chemical and physical properties, chemical classifications and applications, and spectral information associated with each species. The ontology includes provenance and attribution metadata, ensuring the reliability and traceability of data. Most of the information about chemical species are sourced from PubChem and ChEBI data on the respective compound Web pages using a software agent, making OntoSpecies a comprehensive semantic database of chemical species able to solve novel types of problems in the field. Access to this reliable source of chemical data is provided through a SPARQL end point. The paper presents example use cases to demonstrate the contribution of OntoSpecies in solving complex tasks that require integrated semantically searchable chemical data. The approach presented in this paper represents a significant advancement in the field of chemical data management, offering a powerful tool for representing, navigating, and analyzing chemical information to support scientific research.

## 1 Introduction

As Gasteiger^1^ emphasized, cheminformatics boasts a rich history that has left its mark on many chemistry-related fields. Over the years, significant advancements in cheminformatics have been made, with key studies shedding light on techniques and methodologies crucial to contemporary drug discovery and wider applications in chemistry.^2,3^ The ability to use these techniques to extract meaningful insights from data has played a key role in addressing critical global challenges related to food, water, health, energy, and environment. Chemical engineers for example are using data and informatics-based approaches to understand complex systems and develop more efficient and sustainable processes.^4−6^ With the increasing volume and complexity of chemical data, digital tools have facilitated the sharing and dissemination of scientific information, allowing for greater transparency and collaboration within the scientific community.^7^

A large amount of data about chemical compounds are publicly available across many chemistry databases. One of the most comprehensive general public chemistry databases is PubChem,^8−10^ which hosts information on more than 100 million unique chemical structures, collecting data from different sources, including government agencies, university laboratories, pharmaceutical companies, and substance vendors. Chemical Abstracts Service (CAS) is another pivotal database, primarily known for its extensive collection of chemical substances and their associated literature references, providing a unique CAS Registry Number for every chemical substance.^11^ ChemSpider provides a free-to-access collection of compound data sourced from numerous data providers, offering a platform for researchers to search and share chemical structures, properties, and associated information.^12^ Reaxys stands out as a premier chemistry database, offering curated and experimentally verified data on chemical reactions, properties, and related literature.^13^ These databases serve as a key chemical information resource for researchers in many biomedical science areas, including cheminformatics, chemical biology, and medicinal chemistry.

To make use of this vast amount of data, they provide various tools that fulfill criteria for simple and effective searching, where the chemical formula or name of the chemical species can be used as query strings to obtain the information stored in the database.^8,9^ While these tools can sift through enormous amounts of chemical information, they are insufficient if the search needs to fulfill complex criteria or if new information needs to be derived from existing data. For example, the ability to find compounds with a defined set of desirable properties is invaluable for researchers. Simple examples of such properties might include fundamental properties such as boiling point, melting point, solubility, and polarity that play a crucial role in tasks such as the selection of suitable solvents. Automation of more complex tasks such as identification of species in a mixture or determination of the reactivity of chemical species demands access to more specific and complex information such as spectral fingerprints and HOMO–LUMO band gaps. Other databases such as Reaxys offer more specialized tools for complex data filtering. Its structured nature, while efficient for certain tasks, may not be as adept at handling intricate, interconnected, and data explorations. The lack of an open license further restricts its accessibility to a wider audience.^13^

The limitations of traditional databases underscore the need for a more advanced approach to data representation and querying. In this regard, since the landmark publication by Berners-Lee et al.,^14^ the semantic web has been emerging as an increasingly important approach for better access to chemical information, addressing the gaps and challenges inherent in traditional databases. Semantic databases, built upon structured ontologies, offer interconnectedness, standardization, and adaptability. They not only facilitate the integration of diverse data but also excel in handling intricate queries, making them particularly suited to the complex demands of chemical research. The power of the approach has been exploited by numerous tech companies such as Google, IBM, Microsoft, Facebook, and eBay who have revolutionize their products and capabilities using semantic web technologies.^15^ In the context of the pharmaceutical industry, AstraZeneca leads the way on the application of semantic knowledge as a means to drug discovery^16^ and initiatives, such as Open PHACTS, dedicated to leveraging semantic web technologies to create a collaborative platform that enhances cooperation between industry and public organizations.^17^

At the heart of the semantic web are the Resource Description Framework (RDF),^18^ Web Ontology Language (OWL),^19^ and the SPARQL Protocol and RDF Query Language (SPARQL).^20^ RDF provides a means to use machine-readable metadata to categorize and interrogate web resources. It employs a “triple” structure—comprising subject, predicate, and object—that is uniquely identified using Internationalized Resource Identifiers (IRIs).^21^ OWL extends RDF by providing additional vocabulary and a formal logic-based framework for specifying more complex relationships and constraints, thereby enriching the expressiveness of ontologies. These ontologies can be considered to be composed of the terminology box (TBox) for conceptual definitions and the assertion box (ABox) for storing real-world data instances.^22^ SPARQL acts as both the query language and data access protocol, enabling intricate searches within this RDF and OWL-based semantic web.^20^ Building on the foundational technologies of the semantic web, complex queries become not only feasible but also highly efficient.

Integrating chemical data into semantic databases has been previously attempted.^23,24^ However, challenges arise when dealing with data from diverse sources and in varied formats. While current semantic databases have made strides in integrating data on chemical species, the exported information is confined to identifiers and basic properties as for PubChem RDF.^23^ Some databases offer semantic annotations for chemical classification (ChEBI),^25−27^ spectral information (MassBank),^28^ or properties such as boiling points and melting points (Wikidata).^29^ However, there is no semantic database that uniformly provides a comprehensive set of properties that includes physical properties, classification and uses of chemicals as well as spectral information. Additionally, many of these databases lack a SPARQL end point, which restricts the full potential of federated queries in the semantic web context. Moreover, the absence of open licensing in some databases presents an additional hurdle as it restricts the free use and dissemination of the data.

The fragmented and often incomplete state of chemical data also poses significant challenges to machine learning. While machine learning has the potential to revolutionize data analysis, its application is inhibited by its need for large quantities of clean data. Much time is often wasted by simply gathering and cleaning data. This accentuates the importance of accurate and well-structured data.

There are advantages in not just enabling access to large amounts of data but also in allowing machines to read information describing the context of data.^7^ This is also known as making data machine-actionable. The FAIR data principles, which advocate for data to be findable, accessible, interoperable, and reusable, were designed with this very emphasis in mind.^30,31^ The Blue Obelisk Movement laid foundational emphasis on open data and standards in chemical informatics.^32^ Building on such initiatives, the Chemistry Implementation Network and the AdvancedNano GO FAIR Implementation Network further emphasize the growing recognition on the importance of FAIR data principles in contemporary chemistry.^33,34^

The semantic web offers a compelling solution to these challenges. Semantic web ontologies provide a framework to share context-rich data and they pave the way for logic-based representation and integration from diverse data sources that aligns with the FAIR principles .^7^ While they may not necessarily speed up queries or reduce hardware requirements, they have the ability to streamline data discovery and cleaning processes that lead to more efficient data utilization and processing.^14^

The aim of this paper is to demonstrate an approach to address the current challenges in chemical data management and integration and to make chemical data more accessible, interoperable, and actionable for advanced applications in chemical informatics. We do this by updating OntoSpecies, an ontology developed as part of The World Avatar project^35,36^ for the purpose of integrating chemical data via a unified representation of chemical species and associated data. While the initial version of OntoSpecies provided foundational details like empirical formula and molecular weight,^37^ our enhancements incorporate a wide array of identifiers, chemical and physical properties, chemical classifications, applications, and spectral information. The enhancements also permit the inclusion of provenance and attribution metadata for the data describing each species. By assimilating data from PubChem using this enriched ontology and providing access to the integrated data via a SPARQL end point, our long-term aim is to establish an open platform that not only bridges existing divides between various data sources but also leverages the capabilities of semantic web technologies. This paves the way for sophisticated querying and groundbreaking discoveries in chemistry.

## 2 Methodology

In this section, the semantic web representations that currently exist in the domain of general chemistry are outlined along with their current limitations. We then introduce our approach to overcome these limitations, which includes a description of TWA knowledge graph (KG), OntoSpecies implementation schemes, its linking mechanism to other domains within TWA, and the use of an agent to dynamically populate the KG.

### 2.1 Semantic Representation in the General Chemistry Domain

Chemical informatics has a long history of utilizing semantic web technologies. In the 1980s, Gordon et al. introduced a chemistry-specific formal language based on set theory and first-order logic, with the goal of using it in computational contexts.^38^ Another early example of this approach is the Chemical Semantic Web by Murray-Rust and co-workers^39−41^ where Chemical Markup Language (CML) was employed to host the data. CML is an XML language designed to hold most of the chemistry’s central concepts. It was later extended with the development of CompChem by adding computational chemistry semantics on top of the CML schema.^42^ Although the CML schema represents chemical data, covering concepts related to atoms, molecules, reactions, computational chemistry, and spectroscopy, it is not capable of encoding any desired knowledge in such a way that the meaning is wholly preserved. In contrast, semantic web ontologies aim to explicitly describe and relate objects using a formal logic-based representation.

Since OWL became more and more popular in modeling ontologies, more activities of ontology development have been demonstrated in the scientific domain. In the general chemistry and biology domain, the Chemical Entities of Biological Interest (ChEBI) ontology^25−27^ from the European Bioinformatics Institute (EMBL-EBI) is probably one of the most widely used. ChEBI ontology provides knowledge at both a terminology and an assertion level. It is a publicly available and manually annotated ontology, containing approximately 60,000 fully annotated entities, and over 100,000 preliminary (partially annotated) entities, as of the last release.^43^ It provides a comprehensive and well-documented classification of chemical entities. However, ChEBI is manually maintained and, as such, cannot easily scale to the full scope of publicly available chemical data. To address the limitations of manual maintenance, there is a growing reliance on automatic tools like OntoChem^44^ and ClassyFire^45^ designed to systematically classify chemical compounds based on structural features, contributing to the automation and scalability of chemical ontology development.

Other ontologies with scope in the general chemistry domain are the Chemical Information Ontology (CHEMINF)^46^ and the Chemical Methods Ontology (CHMO).^47^ CHEMINF represents the chemical structure and richly describes chemical descriptors and properties, whether intrinsic or computed. It also includes the definition of commonly used software and algorithms, such as the PubChem software library, as well as format specifications for chemical data, such as the MOLfile format specification. Complementary to CHEMINF that covers the computational and theoretical methods, CHMO intends to describe the physical and practical methods such as mass spectrometry and electron microscopy. These ontologies aim to encode the terms, definitions, and logical axioms of chemical information entities. Contrary to ChEBI, CHEMINF and CHMO are just terminological ontologies and do not include any assertion components.

Ontologizing existing databases in RDF format is also demonstrated in the community. ChEMBL RDF^48^ converts data from the ChEMBL database^49,50^ into RDF triples. ChEMBL is a database of bioactive molecules with drug-like properties. The EBI RDF platform encompasses six public life science databases including ChEMBL, UniProt, Reactome, BioModels, BioSamples, and Expression Atlas.^51^ Bio2RDF serves as a mash-up system that integrates publicly available bioinformatics databases to provide interlinked life science data.^52^ PubChemRDF^23^ is the semantic version of the current largest open-source chemical information repository, PubChem.^10^ The PubChemRDF content includes the core chemical information archived in the PubChem compound and substance databases, the semantic relationships between compounds and substances, and the provenance and attribution metadata of substances.

One of the limitations of the RDF version of these databases is that they do not natively support SPARQL end points and protocols. Galgonek and Vondrášek recently addressed this issue by integrating PubChem, ChEMBL, and ChEBI data sets into one database called the Integrated Database of Small Molecules (IDSM) that is accessible through a SPARQL end point that provides an access point to chemical data from different sources.^24^ The IDSM database was created by loading the RDF data downloaded from ChEMBL, ChEBI, and PubChem servers. However, another major limitation is that the RDF version of these data sets includes only basic information on chemical species, i.e., identifiers, geometry, and computed properties like mass and charges. They are generally used for the annotation of chemicals and the navigation of search results. Data on experimental properties or spectral information are currently not reported in any of the mentioned databases. These information are crucial for applications in reaction modeling or lab automation as described later in the paper.

Table 1 shows the main characteristics of the currently developed semantic databases and ontologies in general chemistry. In summary, to our knowledge there is no semantic database that represents data on chemical species at a deep level of knowledge and that is accessible through a SPARQL end point. In this work, with the development of OntoSpecies, we aim to address all the current limitations, with an ontology where the FAIR principles are better realized and a deeper level of knowledge is represented.

**Table 1:**
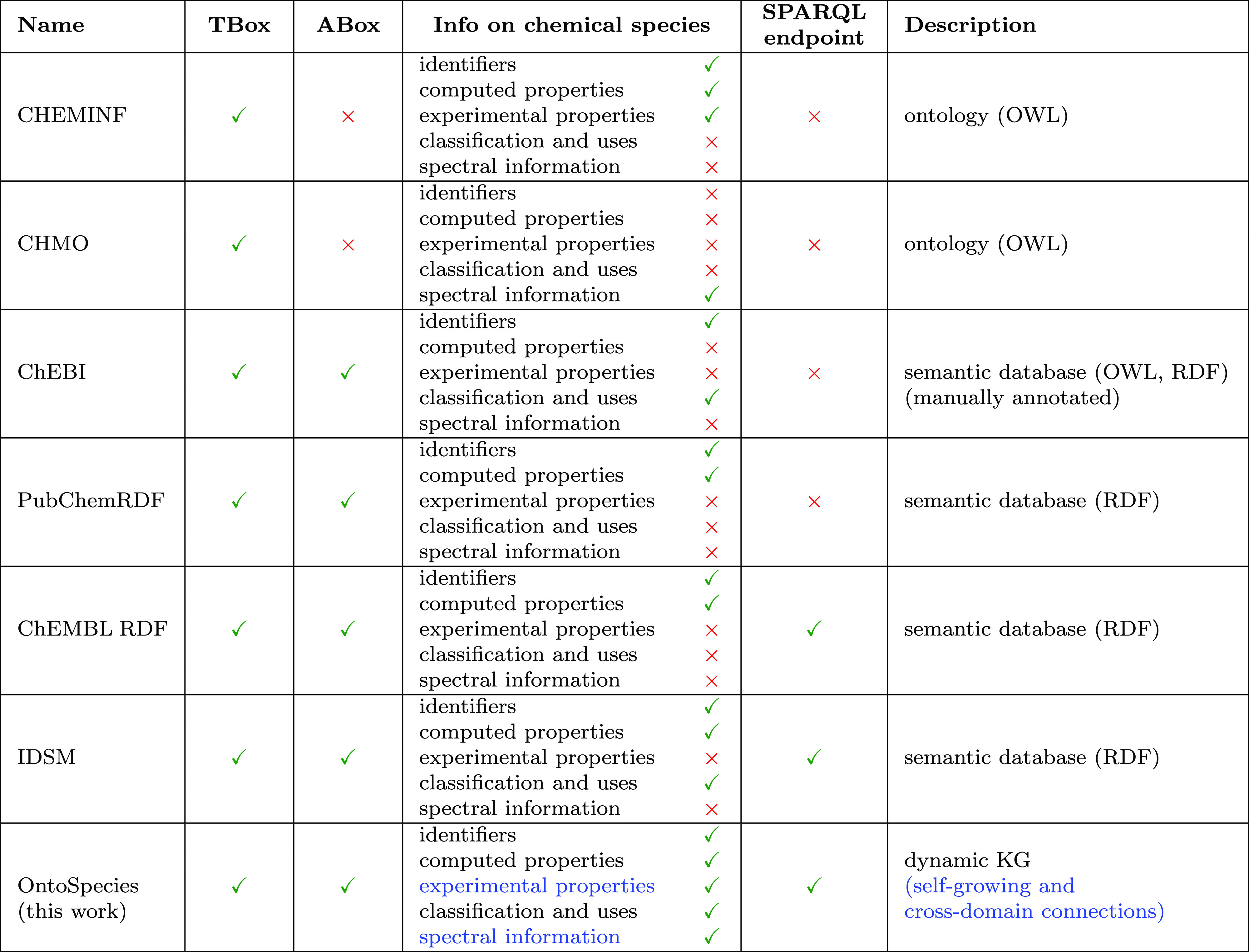
Summary of Key Ontologies and Semantic Databases in the General Chemistry Domain^a^

a Text in blue indicates the novel content and features of OntoSpecies, which distinguish it from the other existing semantic databases.

### 2.2 The World Avatar

The World Avatar project aims to create a digital representation of the real world. The digital world is composed of a dynamic KG that contains concepts and the data that describe the world and an ecosystem of autonomous computational agents. The agents simulate the behavior of the world and continuously update the concepts and data. A KG is a network of data expressed as a directed graph, where the nodes of the graph are concepts or their instances (data items), and the edges of the graph are links between related concepts or instances. KGs are often built by using the principles of Linked Data. In TWA KG, ontologies are used to define the schema or structure of the KG, providing a powerful means to host, query, and traverse data and to find and retrieve related information. The autonomous computational agents are the key aspect of the dynamic nature of the KG. They continuously and independently act on the KG performing various tasks. Such tasks include performing calculations using data in the KG, passing information to other software/users outside of the KG and then taking these results to create new instances in the KG, and updating existing instances in the KG with improved information where appropriate. Agents perform these tasks with the aim of producing a self-growing, self-updating, and self-improving KG. Additionally, an agent can also consist of subagents to answer more complex queries or perform sophisticated tasks.

TWA KG currently includes several ontologies that span a variety of domains. In the process engineering and industrial domains, this includes the well-established OntoCAPE, an ontology for computer aided process engineering that has been integrated into TWA^53^ as well as OntoEIP for describing the functions and interactions underlying eco-industrial parks.^54−56^ In energy and power systems, OntoPowerSys was developed to describe electrical power systems that support industrial plans.^57^ This has also been coupled with OntoTwin, an ontology that allows cross-domain coupling.^58^ Ontologies for semantic smart city planning by utilizing 3D models include OntoCityGML^59^ and the Weather Ontology.^60^ Finally, several ontologies have been developed in the chemistry domain to describe different types of chemical data. It provides ontologies for representation of chemical species (OntoSpecies),^37^ chemical reaction kinetics (OntoKin),^37^ quantum chemistry (OntoCompChem and OntoPESScan),^61,62^ and reaction experiments (OntoReaction—under development).

### 2.3 OntoSpecies

OntoSpecies serves as the core ontology in the TWA chemistry domain. It is an ontology that describes unique chemical species and their chemical properties. In its previous implementation, OntoSpecies was designed to capture basic information about species, such as the empirical formula and molecular weight. It was designed to be linked with other ontologies like OntoKin^37^ and OntoCompChem.^61^ In this work, OntoSpecies has been extended to include a wide range of identifiers, chemical and physical properties, chemical classifications, and applications, plus spectral information associated with each species, and the provenance and attribution metadata. Most of the information about a species are collected from the info archived in the respective PubChem compound web source as explained in “Population and Querying”.

#### 2.3.1 Terminology Component

OntoSpecies TBox containing the full class and relational definitions is available at https://github.com/cambridge-cares/TheWorldAvatar/tree/main/JPS_Ontology/ontology/ontospecies. Each entity is uniquely represented by an IRI. These IRIs are lexically meaningful to help query information from the KG. A set of standardized ontologies for enhanced data integration and interoperability was collected to define the domain specific knowledge, including CHEMINF,^46^ CHMO,^47^ units of measurement (om),^63^ Gainesville Core (gc),^64^ and Simple Knowledge Organization System (skos).^65^ Adoption of these core ontologies helps to ensure that the mapping of chemical information is compatible across multiple semantic web resources. For convenience, prefixed names are used instead of the full IRIs in the following sections that describe the schema and its use cases. The definitions of namespace prefixes used in the paper are summarized in Table 2.

**Table 2:** Prefixes and Corresponding Namespaces of the Ontologies Used in This Work

prefix	namespace
os	<http://www.theworldavatar.com/ontology/ontospecies/OntoSpecies.owl#>
okin	<http://www.theworldavatar.com/ontology/ontokin/OntoKin.owl#>
occ	<http://www.theworldavatar.com/ontology/ontocompchem/OntoCompChem.owl#>
rdf	<http://www.w3.org/1999/02/22-rdf-syntax-ns#>
rdfs	<http://www.w3.org/2000/01/rdf-schema#>
xsd	<http://www.w3.org/2001/XMLSchema#>
owl	<http://www.w3.org/2002/07/owl#>
skos	<http://www.w3.org/2004/02/skos/core#>
om	<http://www.ontology-of-units-of-measure.org/resource/om-2/>
pt	<http://www.daml.org/2003/01/periodictable/PeriodicTable#>
gc	<http://purl.org/gc/>
CHEMINF	<http://semanticscience.org/resource/>
CHMO	<http://purl.obolibrary.org/obo/>

**Figure 1:**
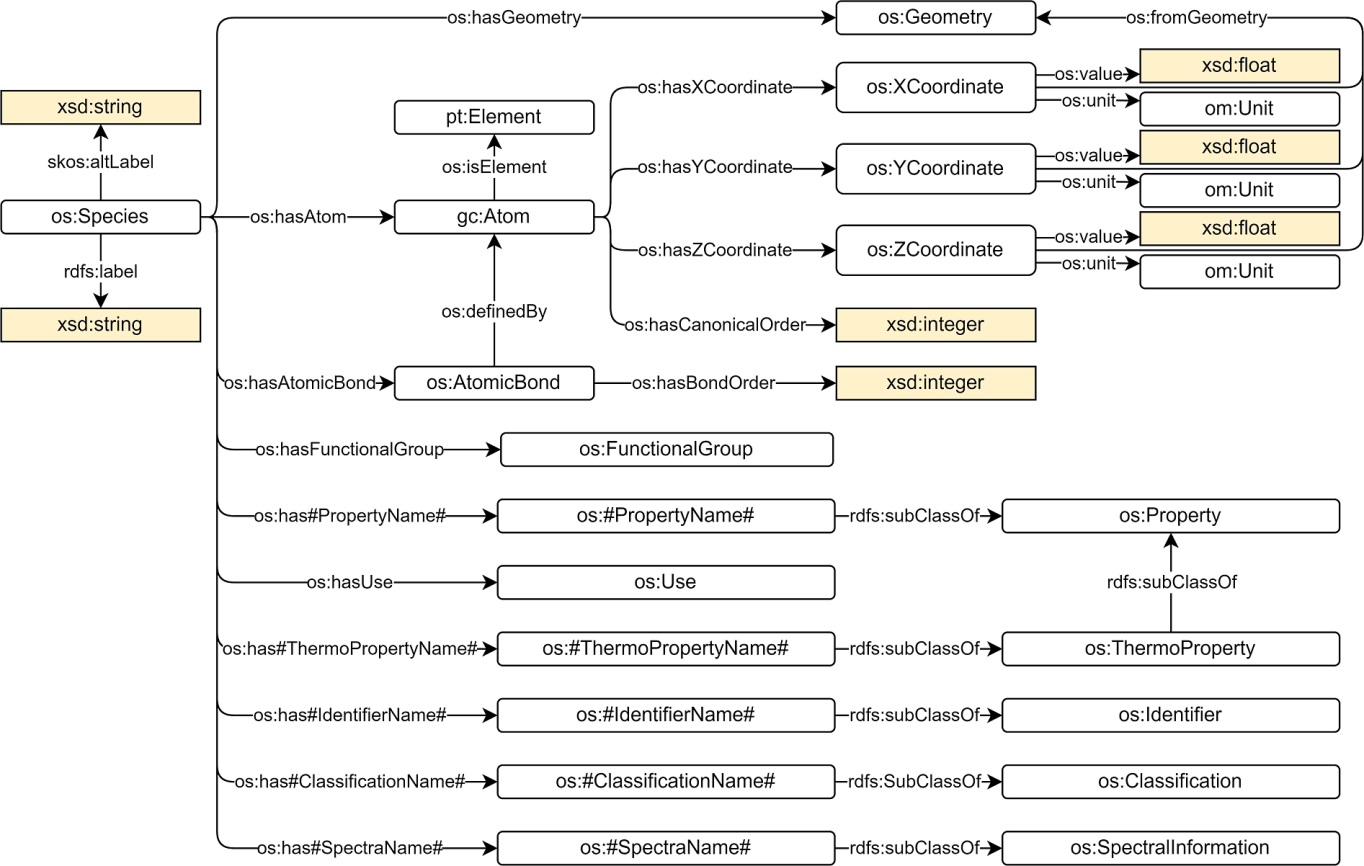
Species concept in the OntoSpecies TBox.

The main class of the ontology is os:Species (Figure 1). A species can be defined as “an ensemble of chemically identical molecular entities”^66^ and can be seen as the equivalent of a PubChem “compound”. Because of the different nomenclature systems that are used by different organizations to refer to a species, we decided to assign its molecular formula as a label to the species (predicate rdfs:label). However, all the synonyms given to a species are also stored as alternative labels (predicate skos:altLabel). This helps querying for a chemical species.

A species is composed of atoms (class gc:Atom). An atom is associated with an element (class pt:Element) in the periodic table through the object property os:isElement. Atoms are connected together by chemical bonds (class os:AtomicBond). A chemical bond has a bond order (predicate os:hasBondOrder) that is a data property of type xsd:integer and is identified by (predicate os:definedBy) the two atoms that participate in the bond. The atom coordinates in space (classes os:XCoordinate, os:YCoordinate and os:ZCoordinate) define the geometry (class os:Geometry) of the species. Atom coordinates are linked to the geometry by the predicate os:fromGeometry. Functional groups (class os:FunctionalGroup), groups of atoms within a molecule that have similar chemical properties whenever it appears in various compounds, are associated with a species through the predicate os:hasFunctionalGroup. Species and elements are also linked to a range of other concepts that are subclasses of one of the following classes: os:Identifier, os:Property, os:Classification, os:Use, or os:SpectralInformation.

**Figure 2:**
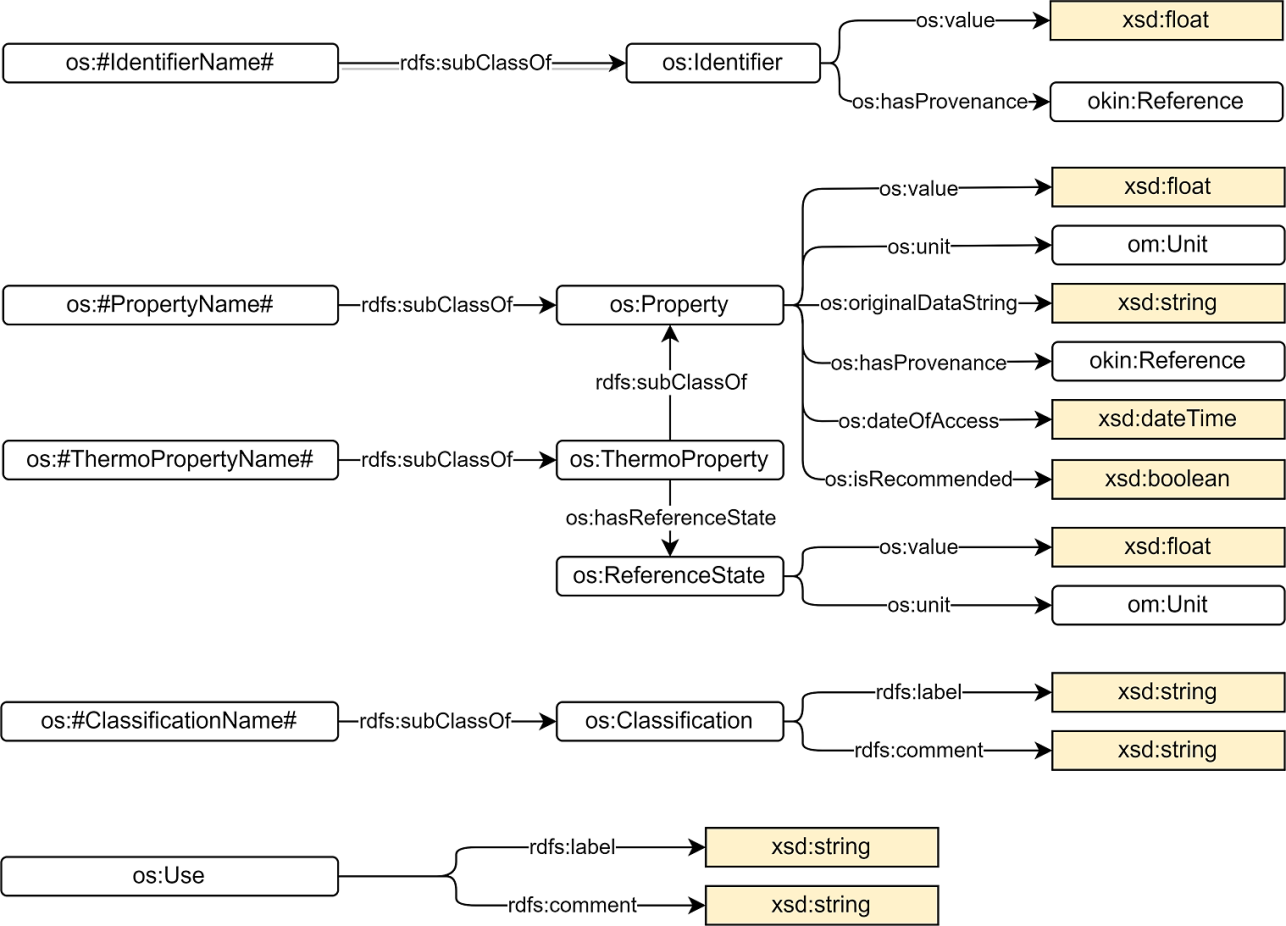
Identifier, Property, Classification and Use concepts in the OntoSpecies TBox.

An identifier (class os:Identifier) provides a way for the identification of a species or element, which in most cases should aim to be unique and easy to use as an unambiguous reference for the chemical entity. The identifier representation consists of triples specifying the value (predicate os:value) and the provenance (predicate os:hasProvenance) of the identifier (Figure 2). Examples of subclasses of the class os:Identifier (defined as os:#IdentifierName# in Figures 1 and 2) are the International Chemical Identifier InChI (class os:InChI), the InChI Key (class os:InChIKey), or the International Union of Pure and Applied Chemistry (IUPAC) Name (class os:IUPACName).

The os:Property class represents a chemical or physical property of a species or element. The property representation consists of triples specifying the value (predicate os:value), the unit (predicate os:unit), and the provenance (predicate os:hasProvenance) of the property (Figure 2). Because most of the properties are collected from the PubChem database, where they are stored in PubChem as strings, the original string (class os:originalDataString) and the date of acquisition (data property of type xsd:dateTime) of the property are also linked to the property. A os:ThermoProperty is a subclass of os:Property that is evaluated at a reference state (class os:ReferenceState). Examples of subclasses of the class os:Property (defined as os:#PropertyName# in Figures 1 and 2) are the molecular weight (class os:MolecularWeight) or the charge (class os:Charge) of the species. Examples of subclasses of the class os:ThermoProperty (defined as os:#ThermoPropertyName# in Figures 1 and 2) are the boiling point (class os:BoilingPoint), density (class os:Density), or solubility (class os:Solubility) of the species.

Chemical species can be grouped by their structure or similar features, by their application and role, or by other factors. They are also classified by organizations around the world. As an example, Globally Harmonized System of Classification and Labeling of Chemicals (GHS) is a United Nations system to identify hazardous chemicals and to inform users about these hazards.^67^ GHS has been adopted by many countries around the world and is now also used as the basis for international and national transport regulations for dangerous goods. In OntoSpecies, we include os:ChemicalClass and os:GHSHazardStatment as subclasses os:Classification and class os:Use to represent the application and role of a species (Figure 2).

**Figure 3:**
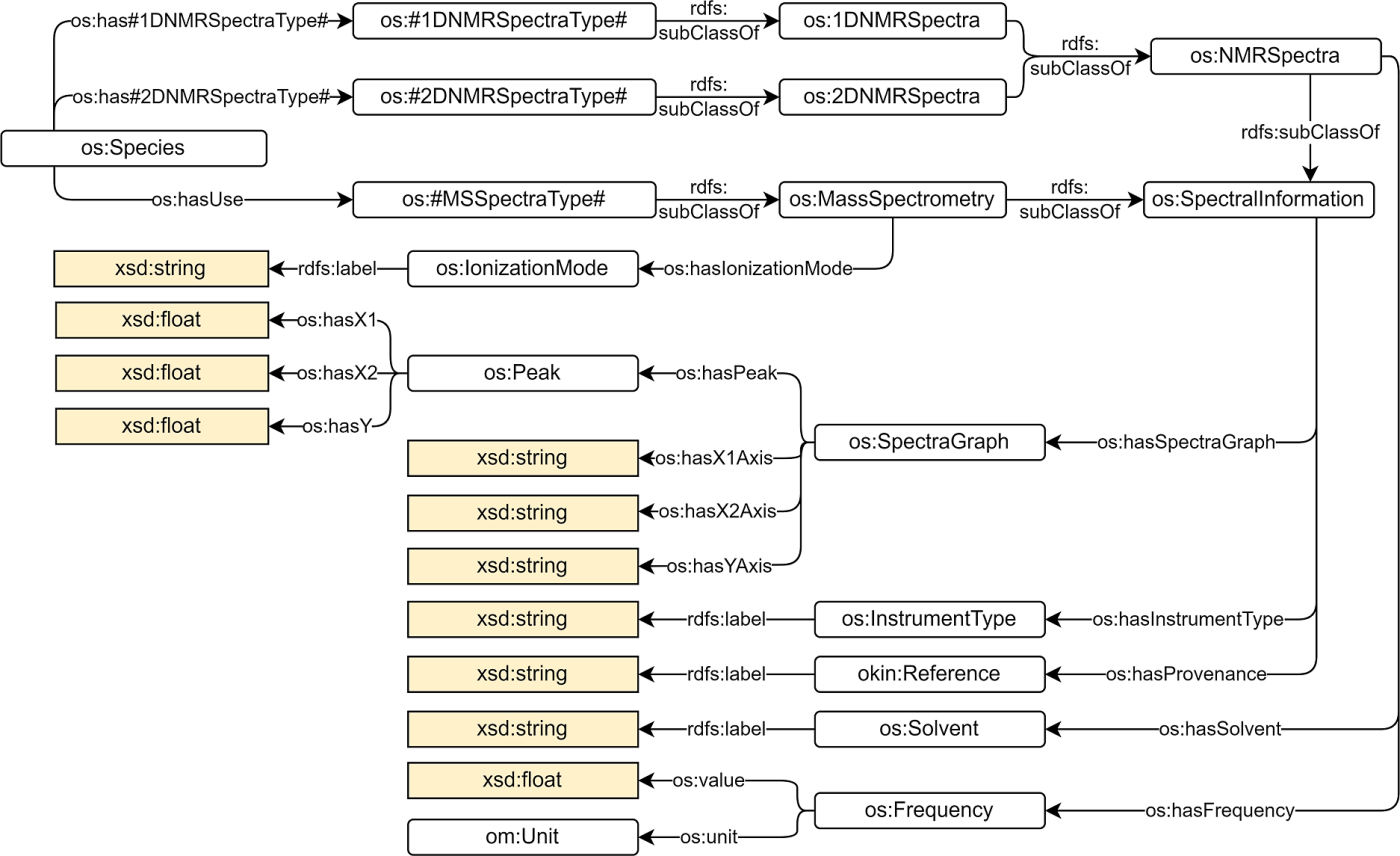
SpectralInformation concept in the OntoSpecies TBox.

Spectral data (os:SpectralInformation) are also linked to the species (Figure 3). At the moment, 1-D and 2-D nuclear magnetic resonance (NMR) (classes os:1DNMRSpectra and os:2DNMRSpectra) and mass spectrometry (MS) (class os:MassSpectrometry) are included in the OntoSpecies TBox. Every spectrum is associated with a graph (class os:SpectraGraph) that shows the peaks (class os:Peak) of the species. Additional information is also recorded like the ionization mode (class os:IonizationMode) for spectra of type os:MassSpectrometry or solvent and frequency (classes os:Solvent and os:Frequency) for spectra of type os:NMRSpectra.

PubChem, as an archive, takes care to preserve the provenance of information. Identifiers and properties are linked to the respective sources (class okin:Reference) through the predicate os:hasProvenance (see Figure 2). The provenance can be given as the URL of a Web page or the DOI of a scientific paper.

A full list of classes in OntoSpecies and their description are reported in the “Supporting Information”. Classes that represent concepts that are already defined in the CHEMINF or CHMO vocabularies are linked to their corresponding class in those vocabularies through the use of the predicate owl:equivalentClass. The association with central vocabularies like CHEMINF or CHMO enables comparison between chemical properties arising from different databases in a standardized fashion thus helping interoperability. The choice to use a lexically meaningful IRI to represent the property instead of directly using its equivalent CHEMINF or CHMO class enhances the reading and querying of data by humans.

#### 2.3.2 Assertion Component

In OntoSpecies ABoxes of the ontology, each entity is uniquely represented by an IRI. The ABox IRIs have the prefix:

PREFIX oskg: <http://www.theworldavatar.com/kg/ontospecies/>

The IRI for a species is assigned as:

oskg:Species_UUID

where UUID is a 128 bit value used to uniquely identify an object or entity on the internet generated at the moment of species instantiation.

The IRIs for instances directly related to a chemical species (e.g., boiling point of a species, molecular weight of a species, or IUPAC name of a species) were constructed based on a combination of their class type name and the species UUID. The IRI also includes an index entry right after the class type name because multiple instances of the same class type might be connected to the same species. For example, the IRI for the boiling point of a species is represented as:

oskg:BoilingPoint_#index#_Species_UUID

where #index# goes from 1 to the number of boiling point instances connected to oskg:Species_UUID.

IRIs of other concepts that are not directly related to a chemical species (e.g., use, unit, or provenance) have the following syntax:

oskg:#ClassTypeName#_UUID

Including the class type name in the IRI makes it easier for a human user to understand what the IRI represents, reducing the errors in the querying stage.

#### 2.3.3 Links to Other Ontologies in TWA

**Figure 4:**
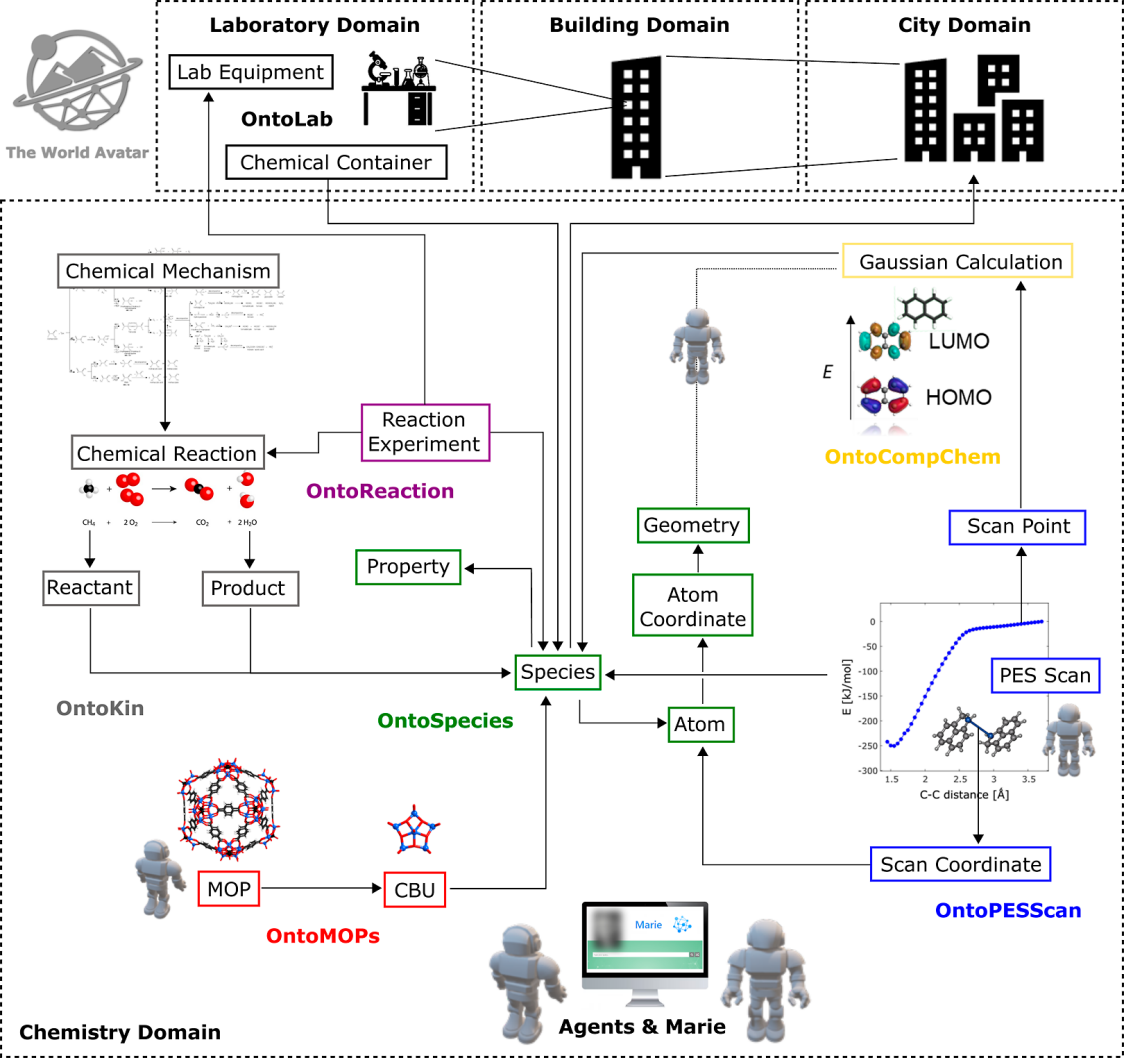
Connection of OntoSpecies to other segments of TWA KG.

OntoSpecies plays a central role in TWA, enabling the linking of species to instances and concepts deriving from other ontologies in the TWA KG chemistry domain (see Figure 4). The other ontologies included in TWA chemistry domain currently are OntoKin,^37^ OntoCompChem,^61^ OntoPESScan,^62^ OntoMOPs,^68^ and OntoReaction (under development).

OntoKin is an ontology that represents reaction mechanisms.^37^ In a chemical process, a reaction mechanism constitutes a set of stoichiometric reactions involving different chemical species. A reaction is described through products and reactants that are further described through different thermodynamic and transport model concepts and identified via OntoSpecies IRIs. OntoKin, in conjunction with OntoSpecies, we can provide a facile and unambiguous comparison between other kinetic, thermodynamic, or transport models reported in the literature.

The OntoCompChem ontology represents the input and output of density functional theory (DFT) calculations, currently mainly focused on molecular systems.^61^ OntoCompChem currently represents single point calculations, geometry optimizations, and frequency calculations. Information such as the final converged self-consistent field energy and the calculated frontier orbitals is also stored for each calculation. For geometry optimizations, the final optimized geometry is represented, while for frequency calculations it stores the zero-point energy correction and a full list of the computed vibrational frequencies. For the representation of potential energy surface (PES) scans, a different ontology has been developed (OntoPESScan).^62^ A PES scan calculation is linked to a number of single point calculations in OntoCompChem as every scan can be seen as a collection of those. The unique identification of bonds and atoms in the OntoSpecies database is used for the identification of geometry changes between calculations.

OntoReaction semantically describes the reaction experiments. Species that take part in the reaction experiment are identified via OntoSpecies IRIs. By assigning different IRIs to species that are based on different isotopes, charges, or spin states, OntoSpecies becomes relevant also for the digital representation of isotope labeling experiments, redox- and electrochemically driven processes, and photochemistry.

OntoMOPs ontology describes metal–organic polyhedra (MOPs).^68^ MOPs are assemblies of organic and metal-based chemical building units (CBUs) resembling the shapes of regular polyhedra. The CBUs are instantiated as species in OntoSpecies.

OntoSpecies is also directly or indirectly linked with other domains in TWA. OntoLab (under development) and related ontologies semantically describe equipment such as reactors, autosamplers, and chromatography devices in order to build complete digital twins of (chemical) laboratories. A direct link between OntoSpecies and OntoLab is the link between a chemical container and the species that it contains while an indirect link is through OntoReaction. The reaction experiment is linked to the species involved in the reaction, and it is also linked to the lab equipment where the reaction experiment is carried out. In the context of digital urban planning, data on chemical species in conjunction with real-time weather data and data on the physical infrastructure can be used to predict the dispersion of pollutants in the atmosphere of urban areas.^69^

Software agents are semantic web services that act upon the KG, collecting, updating, and creating knowledge. Several examples of agents and cross-domain application exist in the TWA chemistry domain. To cite some, for existing DFT calculations (OntoCompChem), an agent instantiates thermal properties (enthalpy, heat capacity and entropy) back to the involved species (OntoSpecies) as well as 7-coefficient NASA polynomials to the reaction (OntoKin).^61^ The species IRI (OntoSpecies) serves as a link between the reaction mechanisms (OntoKin) and the DFT calculation (OntoCompChem). This enables reaction modeling that requires kinetic and thermodynamic factors. The information on molecular geometry (OntoSpecies) can be retrieved and used by an agent as an initial guess of the geometry for quantum chemical calculations (OntoCompChem), and the obtained geometry can be stored as a new geometry connected to the species with the appropriate provenance. A reaction experiment (OntoReaction) can be linked to a reaction mechanism (OntoKin) through the chemical species that take part in the experiment and reaction (OntoSpecies) and agents can do sensitivity analysis and calibration as demonstrated for combustion experiments.^70^ The MOP discovery agents shows that MOPs can be rationally designed, revealing which CBUs can be meaningfully combined without causing undesired strains.^68^

A user-friendly question answering interface, “Marie”, enables users to retrieve information from TWA KG chemistry domain using their natural language, which is then translated behind the scenes into machine readable query. “Marie” makes our infrastructure available to users who are not aware of the knowledge structure in the KG and its different domains.^71,72^

In summary, the interconnection of the ontologies in different domains of TWA in which OntoSpecies plays a central role, as well as the integration with agents and “Marie” has three main outcomes:

1. Facilitate the search for information on a chemical species, reactions, quantum chemistry calculations, and experiments through federated queries (see “Data Enrichment”) or asking questions in natural language to “Marie”.
2. Create new knowledge using information from different subdomains.
3. Bridge the gap between molecular-scale chemistry and real-world macro-scale phenomena.

### 2.4 Population and Querying

**Figure 5:**
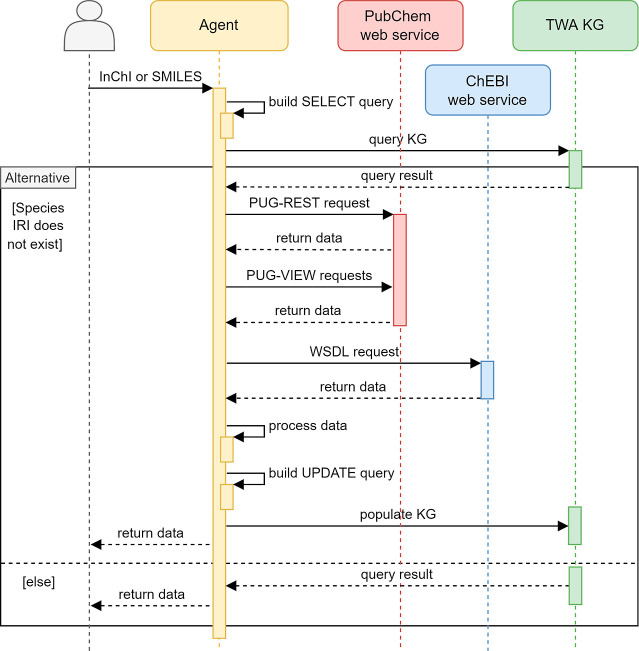
UML sequence diagram of the software agent.

To help with creating and uploading entries to the KG, a software agent has been developed to request data from chemistry databases and create the necessary OWL files for the OntoSpecies instances, streamlining the population process. The agent is freely accessible at https://github.com/cambridge-cares/TheWorldAvatar/tree/main/Agents/PubChemAgent. The current version requests information from PubChem and ChEBI, but it can be easily extended to other chemical databases. The agent can be used locally or as a simple web application. Its UML diagram is reported in Figure 5.

The agent receives an InChI or SMILES identifier of a species as input and builds its queries accordingly. In the first step, it checks if the species is already instantiated. If this is not the case, the agent requests the relevant information from PubChem and ChEBI.

PubChem provides two representational state transfer (REST) interfaces as programmatic access routes to PubChem data: PUG-REST^73−75^ and PUG-View.^76^ PUG-REST retrieves information computed by PubChem while PUG-View gives information collected from other sources, including annotations. PUG REST offers the same information as PubChem RDF, but it allows us to make more targeted requests for data, specifically for the species we wanted to instantiate in our study. This approach allowed us to streamline the data retrieval process and work with a more manageable subset of the information, which was more aligned with the scope of our research. We decided to use PUG-REST to obtain information on identifiers and computed properties, by PubChem and PUG-View to obtain experimental properties, chemical classes, uses, spectral information, and synonyms of species that are not accessible by a PUG-REST request. While computed properties are reported using the same format (value plus unit), the experimental properties are reported as strings from different sources and with different syntax and units. For example, if we look at the solubility entries for ethanol in PubChem, the following strings are listed on the compound Web page:

- Greater than or equal to 100 mg/mL at 73 °F (NTP, 1992).
- 1,000,000 mg/L (at 25 °C).
- In water, miscible/1 × 10 + 6 mg/L/at 25 °C.
- Miscible with ethyl ether, acetone, and chloroform; soluble in benzene.
- Miscible with many organic solvents.
- 1000.0 mg/mL.
- Solubility in water: miscible.
- Soluble in water.
- Miscible.

In order to turn these strings into quantitative values that can be assigned to the data property in OntoSpecies, we used the unit-parse Python package.^77^ For each string, a parser translates the string in cleaned and parsed quantities and units that are then converted into SI units. This enables easy comparability of properties among different species. If a numerical value is not detected, then the property string is discarded by the agent. If multiple values for the same property are collected from different strings, all of the values are saved in different instances of the property class. A preferred value is also selected by first grouping approximate equivalent quantities and then discarding entries with faulty units or far-off values. The preferred value is then stored in a new instance of the property class and the value “True” is assigned to the data property os:isRecommended (see Figure 2) and the label “PubChem agent” is assigned to its provenance.

Classification of species is taken from the ChEBI database through Web Services Description Language (WSDL) request. Chemical classes in ChEBI are organized in a tree structure, also known as a hierarchy, based on the chemical structure of the entities. The lower levels of the hierarchy become more specific with each category describing a smaller subset of chemical entities. This hierarchical structure allows for easy navigation and categorization of chemical entities based on their properties and characteristics.^26^ When a new chemical class from ChEBI is instantiated in OntoSpecies, the agents check if all the parent classes (upper level classes) are already instantiated. In case they are not, the agent creates an instance of the parent class and checks for its parent classes. The process is repeated until the full ChEBI classification hierarchy is imported into OntoSpecies. Data on functional groups along with their hierarchy are also collected from ChEBI.

The collected data from PubChem and ChEBI for a chemical species is then put in the form of a SPARQL update and instantiated in the KG. As of April 2023, the OntoSpecies KG includes more than 35,000 species, and new species along with their properties are continuously instantiated.

Shapes Constraint Language (SHACL)^78^ validation has been performed on the generated triples to ensure not only accurate data importation but also strict adherence to structural and semantic standards (see “Data Validation Using SHACL” in the “Supporting Information”).

Information on how to access OntoSpecies can be found at https://theworldavatar.io/chemistry/documentation/ontologies/ontospecies.

## 3 Results and Discussion

### 3.1 Data Access via Complex Queries

The ontological format permits complex queries, easy data analysis, and visualization. This can be used to compare chemical properties of similar compounds, find compounds with required characteristics, as well as automate laborious data gathering from research activities. To demonstrate the strengths and capabilities of the extended OntoSpecies, several use cases are presented in this section, along with their corresponding SPARQL queries. The successful application of software agents postprocessing the obtained data and dynamically expanding the KG is also showcased.

#### 3.1.1 Use Case 1: Reproduce, Monitor, and Investigate Trends in Chemical Properties

Different classes of molecules and compounds follow specific trends in their physical properties, e.g., boiling temperature. These trends can be monitored against a well-defined parameter relevant to the structure of the molecules such as the carbon chain length in aliphatic hydrocarbons. Classical examples are homologous series of organic molecules such as straight-chain alkanes, alkenes, and alcohols.^79^ The OntoSpecies ontology provides the infrastructure for time-efficient queries of compounds’ physical properties and structural information to illustrate trends.

**Figure 6:**
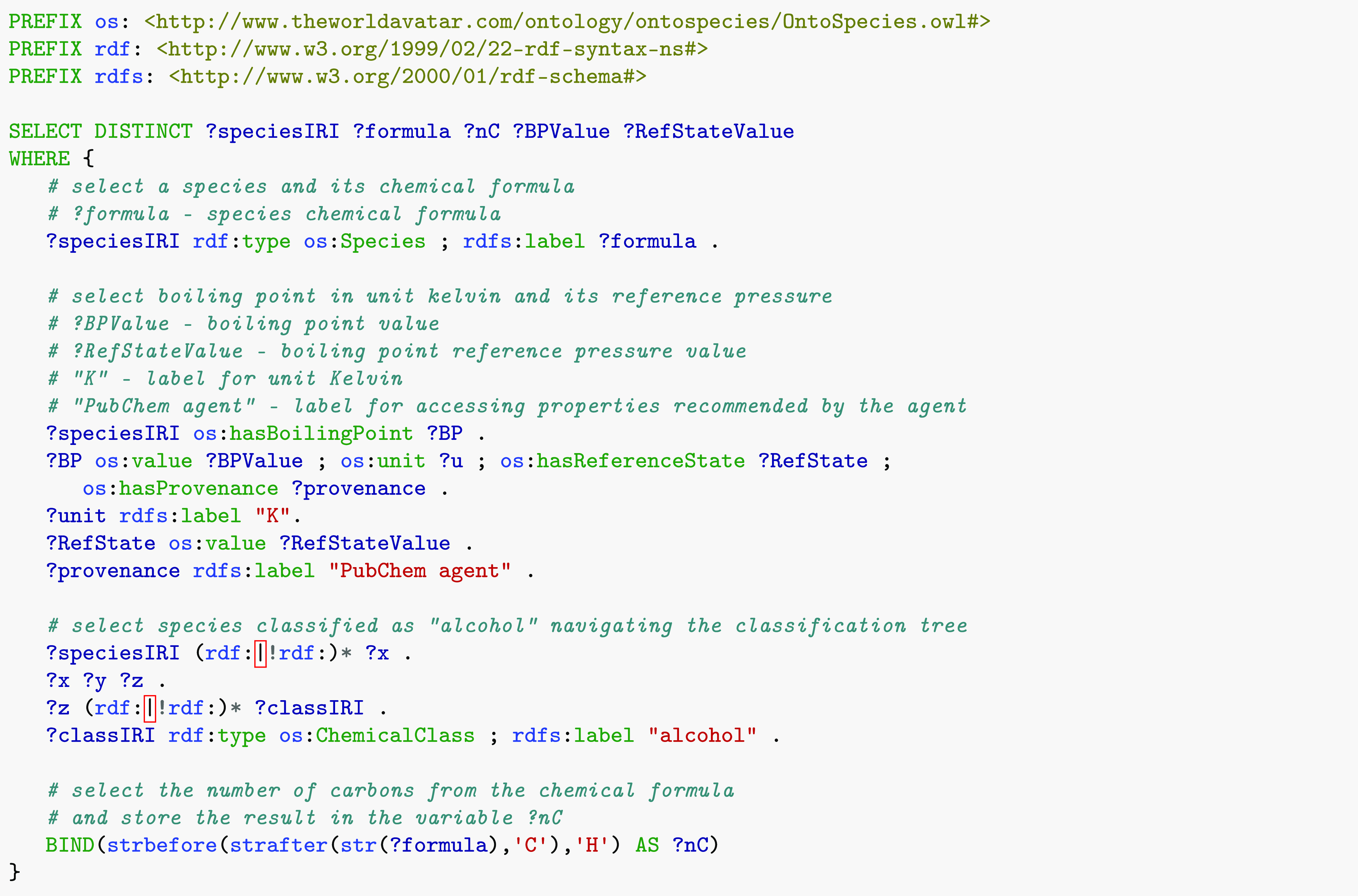
SPARQL query that returns the number of carbon atoms, boiling point, and the boiling point reference pressure of alcohols.

**Figure 7:**
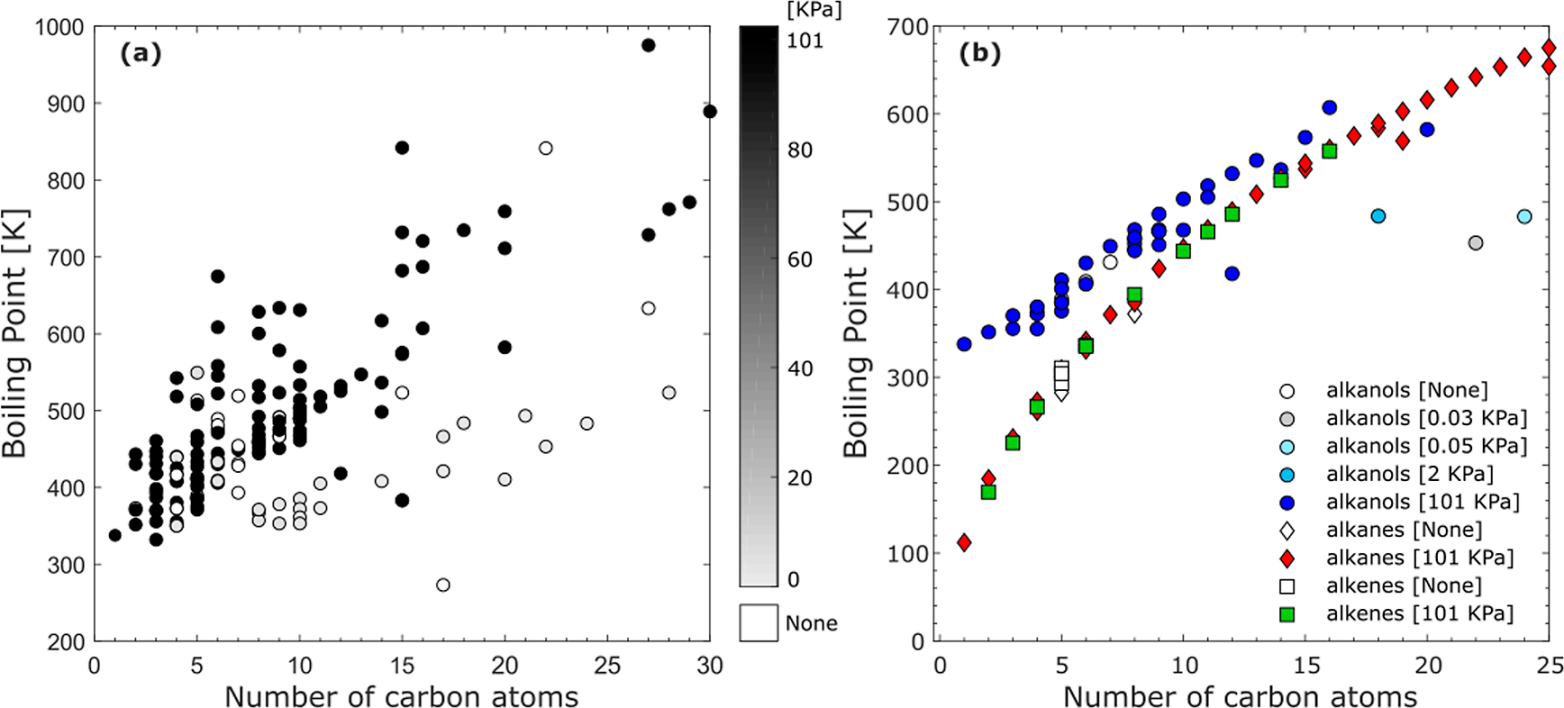
Illustration of boiling point trends for different classes of aliphatic organic molecules in comparison with increasing carbon chain length generated by OntoSpecies queries: (a) alcohols and (b) alkanes, alkenes, and alkanols. Different colors correspond to different reference pressures.

Figure 6 shows an SPARQL query that selects the boiling point temperature of all species classified as alcohols. The query returns the number of carbon atoms, boiling point temperature in K, and the boiling point reference pressure in kPa for 214 different alcohol species. Figure 7a shows the boiling point temperature versus the number of carbon atoms for the alcohols (circles).

Filtering species only based on broad chemical classes such as alcohols might not lead to a well-defined trend as shown in Figure 7a. This is caused by differences among species in the same chemical class that may have additional functional groups or a very different structure (e.g., primary alcohol, secondary alcohol, or presence of double bonds). Additional restrictions can then be applied. As an example, alkanol species are alcohols that have a chemical formula of the type C_*x*_H_2*x*+2_O. This subclass of alcohols can be selected by adding a filter on the chemical formula to the query reported in Figure 6. The results are plotted in Figure 7b (circles), and we can see that they follow a more defined trend than their parent class (alcohols). Figure 7b also shows the boiling point trend for alkanes (diamonds) and alkenes (squares).

The application of the chemical class taken from ChEBI in combination with the boiling points provided by PubChem demonstrates the interoperability of the TWA approach. Moreover, the use of additional filters such as sum formulas shows the capability of adding knowledge that is not included in the data sources such as the concept of an alkanol that does not exist in ChEBI (see “Data Enrichment”). Trend charts can also be helpful in inferring unrecorded properties like the reference state, which is missing in some of the data. For example, in Figure 7b, white diamonds and squares (boiling points of alkanes and alkenes with no given reference pressure) are well aligned with green squares (alkenes at 101 kPa) and red diamonds (alkanes at 101 kPa) which suggests that the none-recorded reference pressures are highly probable to be 101 kPa. Similar analysis for the white circle (alkanols with no given reference pressure) suggests that these data are collected at the reference pressure of 101 kPa too.

More complex and lesser-known trends were also investigated. For example, the size dependence of alkanes’ ionization energies is still a topic of ongoing research.^80^ The trend observed via experimental results by Bakulin and Orekhov^80^ to develop an underlying model were qualitatively reproduced by data available via PubChem (see Figure S1 in “Supporting Information”).

Finally, the ability to easily investigate properties of classes also enables the prediction of unknown values. In the simplest case, unknown values of a molecule that is part of a group with a well-known trend along a single variable (e.g., boiling points of large alkanes) can be extrapolated (see “Data Enrichment”). However, also multivariate models based on machine learning or group contributions can also be developed.

#### 3.1.2 Use Case 2: Selection of Suitable Solvents Based on Multiple Criteria

The selection of suitable solvents is a key challenge in experimental chemistry. Due to the importance and complexity of this multiobjective task (especially considering heightened focus on sustainability), many guidelines and tools have been developed^81−83^ to assist in the selection of solvents for a given reaction or separation. Most selection criteria can be summarized in four categories:

1. Technical considerations: stability at operating condition (e.g., boiling point), miscibility with solute (e.g., solubility in water), etc.
2. Separability for detection (e.g., different molecular weight for MS detection, different NMR peak locations) or further processing (e.g., different boiling points for crystallization).
3. Sustainability considerations: efficient solvent utilization, recovery and reuse (e.g., boiling point for ease of distillation and separability of mixtures), and potential environmental, health, and safety impacts (i.e., acute toxicity, flash point for risk of ignition).
4. Economic considerations (e.g., raw material cost, and cost related to disposal, recovery, abatement, and liability).

Criteria of all four categories are mostly related to quantitative properties that are represented within OntoSpecies (see “OntoSpecies”). Complex queries with OntoSpecies therefore enable selection or preselection of solvents based on multiple criteria. As an example, a query can be used to assist chemists and process engineers in decisions regarding solvent separability. Solvent exchanges through distillation are quite common in pharmaceutical syntheses, and not all solvent mixtures are easily separated.

The SPARQL query that selects all species that can be used as cosolvents for propan-2-ol and are classified as alcohols is shown in Figure S2 in “Supporting Information”. We decided to use alcohols because they are generally more desirable than solvents in other classes (e.g., bases or ethers class).^83^ We applied two selection criteria:

1. The cosolvent boiling point needs to be 15 K lower or higher than the propan-2-ol boiling point to ensure ease of separation between the two solvents by distillation.
2. Exclude cosolvents with high potential health impact. To do so, we removed all the species that have GHS safety statement related to risk of cancer and risk for unborn child.^81^

Applying the first criterion, only five alcohols are reported by the solvent selection guide by Curzons et al.^83^ as suitable cosolvents for propan-2-ol to enable easiest separation by distillation: 2-(2-butoxyethoxy)ethanol, 2-methoxyethanol, butan-1-ol, ethylene glycol, and methanol. However, a list of 49 solvents is returned querying OntoSpecies, including the five alcohols selected by the solvent selection guide by Curzons et al.^83^ If we apply both the first and second criteria, then a list of 45 species is returned by the query. Undesirable species, like 2-methoxyethanol, were removed from the selection as they are considered dangerous for human health as also reported by GSK and Sanofi’s selection guides.^81^ The list of compounds is reported in the “Supporting Information”. This indicates that our approach is evidently capable of assisting in solvent selection considering solvents that are included in existing solvent selection guides while also considering potential new solvents that are not commonly included in these guides.

It is worth noting that some of the species might be too costly to be feasible as a solvent. Cost considerations are excluded from our selection because raw material costs are currently not present in OntoSpecies and their inclusion will be the subject of future work. However, some cost considerations can still be taken into account indirectly—e.g., by selecting solvents with a boiling point that is not too high (*T*_b_ < 150 °C) to reduce the cost of solvent recovery by distillation. A list of only 13 species is returned if we include the third criterion in the selection (see column “Criteria 1–2–3” of Table S2 in “Supporting Information”).

#### 3.1.3 Use Case 3: Identification of Species in Unknown Mixture Based on the NMR Spectrum

In chemical engineering and related research fields, researchers often need to use tabulated data from Web sites or publications, which involves tedious searches and manual copying of data into their own tables. This especially applies to the analysis of unknown compounds via spectroscopy. Nuclear magnetic resonance (NMR) spectroscopy is based on the nuclear spin exhibited by some nuclei such as ^1^H. Based on overall molecular structure, different hydrogen atoms in a molecule exhibit different resonance frequencies at which its spin flips to align with an external magnetic field.^79^

**Figure 8:**
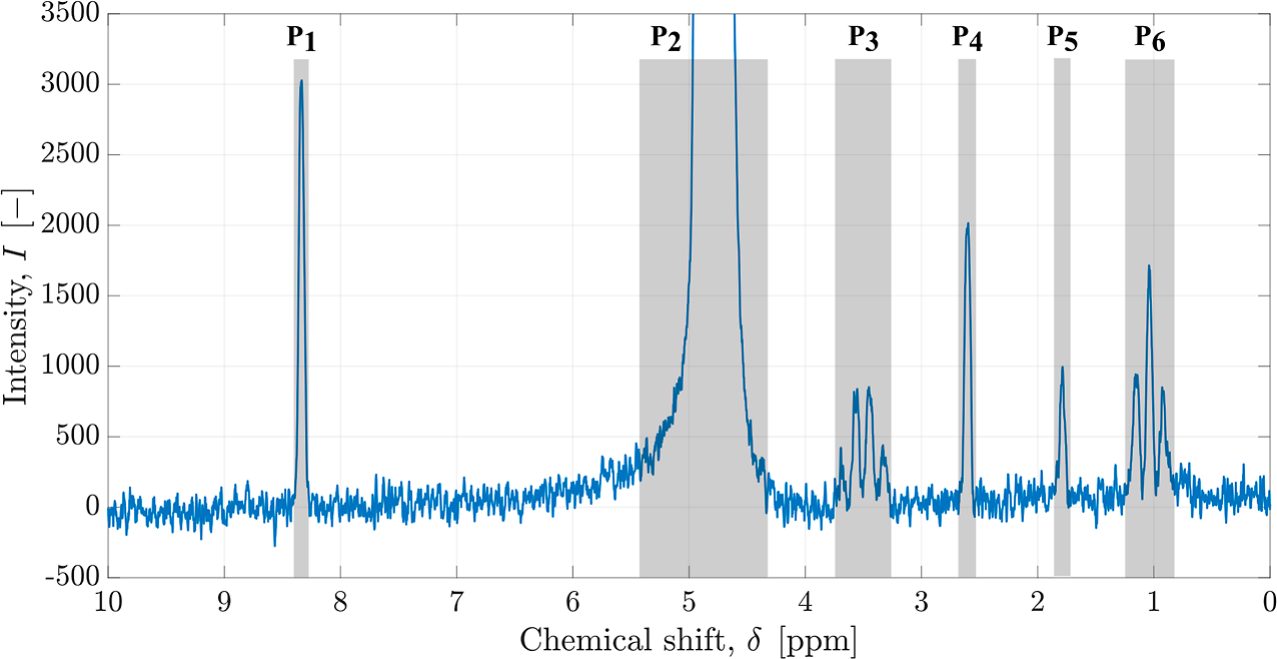
^1^H NMR spectrum on a sample of catholyte after *t* = 20 min of electrocatalytical CO_2_ reduction with internal dimethyl sulfoxide (DMSO) standard adapted from Rihm et al.^84^ The six identified peaks and their respective ranges that were defined are marked in gray.

In an NMR spectrum, the detected intensity is shown on the vertical axis, and the shift of the nuclei’s resonance frequency is indicated on the horizontal axis. In ^1^H NMR, different protons exhibit distinguishable resonance frequencies because they are located in different areas of the molecule and exhibit different interactions with other nuclei, resulting in a “chemical shift” compared to a reference standard. As an example, Figure 8 shows the ^1^H NMR spectrum of a catholyte sample taken from an electrochemical reactor synthesizing a variety of organic molecules from carbon dioxide and water.^84^ As minor products of this reaction are partially unknown, the NMR spectrum is used to detect liquid products of the electrochemical CO_2_ reduction.

To identify the species, expected chemical shifts of all possible products were collected manually, summarized in a table and then compared to the experimental data for every species.^84,85^ This process can be time-consuming and introduces bias by leaving some species out of the analysis. For this reason, there have been many efforts to automate spectral deconvolution and analysis,^86−88^ many of which rely on a standardized procedure or manufacturer-specific software.^86^ Lately, some impressive results were achieved with the help of machine learning tools, in particular deep learning techniques.^87^ Training of these models requires massive data sets (e.g., derived from PubChem^87^) and often additional knowledge to predict shifts and multiplicities—for example, using an assignment algorithm as described by Howarth et al.^88^ As we have access to known NMR spectra of thousands of components from PubChem as well as structural and classification data from other sources that can be queried, OntoSpecies and TWA enable a dynamic approach to automated spectral analysis.

In an initial attempt, we developed an agent to assist analysis of the NMR spectrum in a semiautomated manner: First, the possible species that can be produced in the electrochemical reaction are detected querying for all the species with chemical formula C_*x*_H_*y*_O_*z*_ and with *x* < 5 and *z* < 10. An additional filter on the boiling point (*T*_b_) is added to remove all of the species that are not expected to be found in the liquid phase at room temperature. The selected *T*_b_ = 15 °C is lower than then the experimental temperature (*T* = 25 °C) to ensure all species that might partially be in the liquid phase are included. The respective SPARQL query is shown in Figure S3 in “Supporting Information”. The IRIs of 187 species are returned as a result of the query.

The IRI of each species is then used in a second query to get the ^1^H NMR spectral information (peak shift and intensity) as shown in Figure S4 in “Supporting Information”. A filter is used in the query to select only the first ^1^H NMR spectra added to OntoSpecies from the PubChem record. However, different kinds of filters that select the spectra according to the solvent used or frequency can be constructed instead. 79 species out of the 186 species have recorded ^1^H NMR spectral information.

The recorded ^1^H NMR spectrum as shown in Figure 8 presents six main peaks. The chemical shift (δ_*i*_), multiplicity, and expected species contributing to each peak P_*i*_ are reported in Table 3. The DMSO peak was easily identified by its known chemical shift of 2.6 ppm used as the reference point for all other species. The large peak at 4.9 ppm corresponds to the water solvent. A software agent is used to detect which among the 79 species have peaks with chemical shift in the range [δ_*i*_ −0.2 ppm, δ_*i*_ +0.2 ppm], excluding the DMSO chemical shift. Prior to this, all peaks with an intensity below 20% of the species’ highest peak were discarded. Twelve possible species are selected by the agent. We can narrow down the selection even further by considering the peak multiplicity (singlet, triplet, quartet) and counting reported peaks within the given range of the chemical shift. The final result obtained from the agent is a list of four possible main species produced in the electrochemical reaction: formic acid, acetic acid, ethanol, ethoxyethane.

**Table 3:** Chemical Shifts and Multiplicity of the Peaks Seen in the ^1^H NMR Spectrum in Figure 8 and the Expected Species Contributing to Each Peak Returned as a Result from the Software Agent

peak ID	chemical shift, δ [ppm]	multiplicity	possible species
1	δ_1_ = 8.35	singlet	formic acid (HCOOH)
2	δ_2_ = 4.9	singlet	water (H_2_O)
3	δ_3_ = 3.51	quartet	ethanol (CH_3_CH_2_OH), ethoxyethane ((C_2_H_5_)_2_O)
4	δ_4_ = 2.6	singlet	DMSO ((CH_3_)_2_SO)
5	δ_5_ = 1.83	singlet	acetic acid (CH_3_COOH)
6	δ_6_ = 1.04	triplet	ethanol (CH_3_CH_2_OH), e thoxyethane ((C_2_H_5_)_2_O)

As shown in Table 3, formic acid and acetic acid can be unmistakably identified, while the triplet and quartet could be caused by ethanol or ethoxyethane. At this point, additional reasoning has to be done: ethanol is a much more likely candidate as it is a known product of electrochemical CO_2_ reduction^85^ and despite its much higher boiling temperature was identified in the gas phase.^84^ This reasoning could also be implemented via a software agent, but this is not feasible in this example. Finally, some minor products might exhibit small signals in Figure 8 that overlap with peaks P_3_ and P_6_ as their signal pattern does not appear completely symmetrical. Another software agent was searching for such species and returned a list of possible subproducts as shown in the “Supporting Information”.

### 3.2 Data Enrichment

The enrichment of databases is fundamental to maintain them, as well as the consistency and accuracy of the data.^7^ In this section, we demonstrate the data enrichment capabilities of our approach with a few examples.

#### 3.2.1 Case 1: Assign Properties from Species Similarities

As mentioned previously, the ability to easily investigate properties of classes can also enable prediction of unknown values. In the simplest case, the unknown properties of a molecule that is part of a group with a well-known trend along a single variable can be extrapolated. For example, boiling points of alkenes as a function of number of carbon atoms follow a defined trend as shown in Figure 7. However, in OntoSpecies there are species classified as alkenes, where the boiling point is not reported. The remaining values can be extrapolated by fitting the trend of alkene boiling points taken from OntoSpecies/PubChem with a cube root function using an agent.

**Figure 9:**
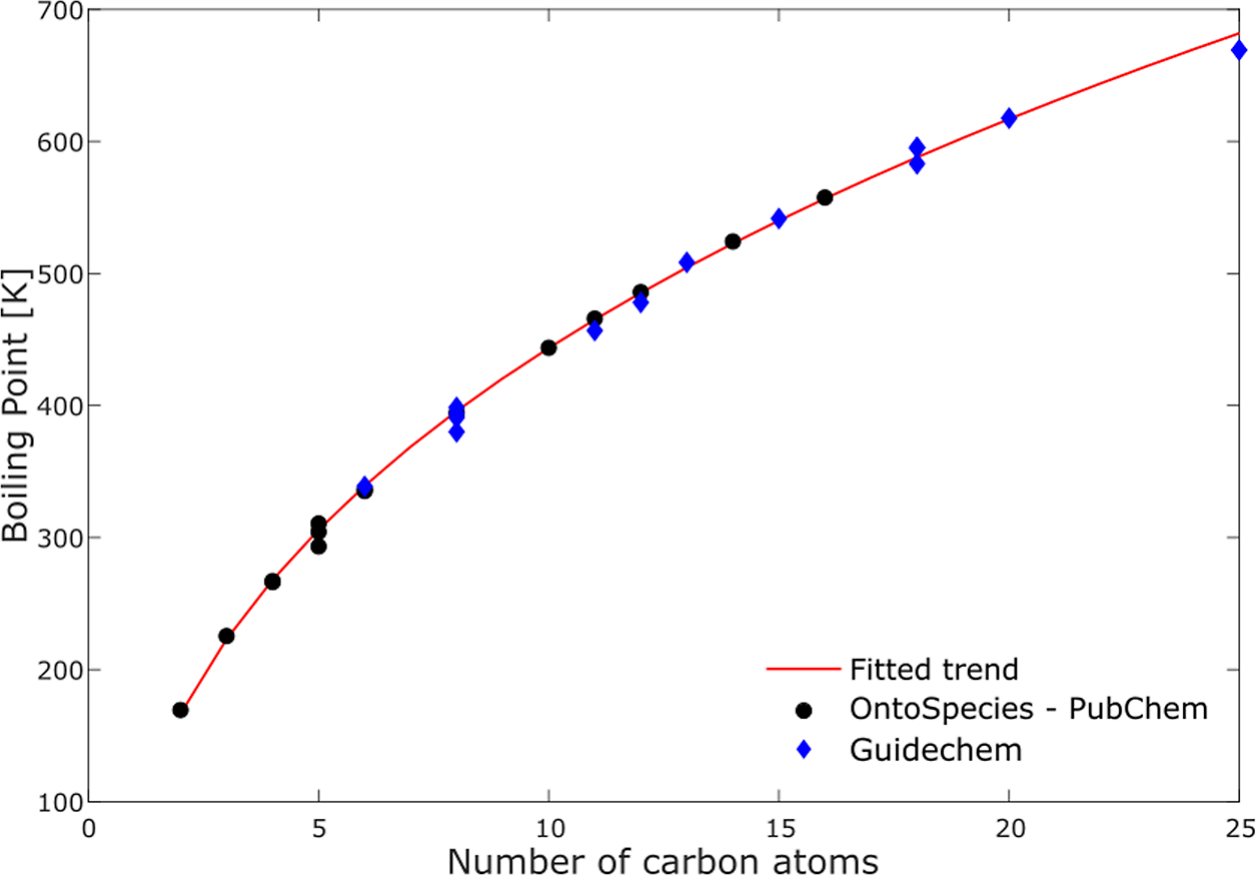
Boiling points for alkanes as a function of the number of carbon atoms. Black circles and blue diamonds show the experimental values taken from OntoSpecies/PubChem or Guidechem databases, respectively. Red line represents the fitted trend using a cube root function.

The extrapolated boiling point trends (red line in Figure 9) have been compared with experimental values taken from Guidechem database^89^ (blue diamonds in Figure 9) and they give a good prediction of alkene boiling points as shown in Figure 9. Obtained values are listed in Table S4 in “Supporting Information”.

The extrapolated boiling points can be instantiated and assigned to the respective species in OntoSpecies by the agent using an SPARQL update query.

#### 3.2.2 Case 2: Find Molecules That Miss Classification Tags

Classification in OntoSpecies is taken from ChEBI. ChEBI is manually maintained and, as such, cannot easily scale to the full scope of public chemical data. Many compounds in PubChem databases are not reported in ChEBI so they miss the classification tag. As an example, if we query for species that are classified as “alkene” in OntoSpecies, only 35 species are returned. However, if we consider that alkenes are molecules with sum formula C_*x*_H_2*x*_ that also have one double bond and we apply this constraint in our query we find that 78 species miss the “alkene” classification tag. We manually checked that all the returned species (e.g., pent-2-ene, dec-4-ene, etc.) actually belong to the alkene class of molecules. The chemical class IRI representing “alkene” molecules can then be linked to these species using an SPARQL update query (Figure S5 in “Supporting Information”). This improves the reusability and findability of the data, and it also allows inference through the navigation of the ChEBI hierarchy (i.e., a species is an alkene and therefore a hydrocarbon).

We can also check for inconsistency of data. We indeed noted that there is a compound that does not have chemical formula of the type C_*x*_H_2*x*_, but it is tagged as “alkene” in ChEBI: 7,11,15-trimethyl-3-methylene-hexadec-1-ene with a sum formula of C_20_H_38_ which is indeed an alkadiene as it exhibits two double bonds.

#### 3.2.3 Case 3: Introduce New Concepts of Chemical Classes

Some chemical classes are not included in ChEBI, such as the “alkanol” class. Alkanol species are alcohols that have a chemical formula of the type C_*x*_H_2*x*+2_O. The use of ChEBI classification in combination with a filter on the chemical formula as previously done in “Data Access via Complex Queries” permits the selection of such species using an SPARQL query. The alkanol species returned by the query (e.g., ethanol, propan-1-ol, propan-2-ol, etc.) are manually verified to ensure the accuracy of the classification. A new chemical class labeled “alkanol” can now be linked to these species and assigned as subclass of the class labeled “alcohol” using an SPARQL update query. This demonstrates the ability of our approach to add knowledge that is not included in the data sources.

#### 3.2.4 Case 4: Check Coherence of Data Reported in PubChem

PubChem gathers data from different sources. Identification of discrepant data in aggregated databases is a key step in data curation and remediation. PubChem data are stored in the form of strings. To get data from PubChem and put it in an ontological format, an agent is used to process the strings and get the numerical value and its related unit. Different strings for the same properties might be present in the PubChem record; therefore, the agent also selects a recommended value as described in “Population and Querying”. This exercise also serves to identify properties of a species with discrepancies between reported values. Using OntoSpecies this can be done using a query that identifies species with a specific property value that is 20% higher or lower than the recommended value. The query example for boiling point is reported in Figure S6 in “Supporting Information”.

For example, methyl acetate is selected as a species with discrepancies in the boiling point values. The list of boiling points of methyl acetate taken from PubChem is reported in Table 4. If we look at the boiling point values, we see that one is much higher than the other ones (red entry in Table 4), due to most likely a typo in the original string. All the other values are similar to the one picked by the agent as the recommended value (blue entry in Table 4). As PubChem collects its data from several external sources, properties are linked to their respective sources through the predicate os:hasProvenance. We can then find out that the discrepant entry is taken from Human Metabolome Database (HMDB) database (http://www.hmdb.ca/metabolites/HMDB0031523). Preserving the provenance of information is crucial for ensuring the integrity, reliability, and usability of information over time. It helps to promote transparency and to facilitate collaboration and sharing.^7^

**Table 4:** Boiling Point Values Are Listed in PubChem for Methyl Acetate^a^

os:BoilingPoint			
index	os:originalDataString	os:value	os:unit
1	134.4 °F at 760 mmHg (NTP, 1992)	330.03888	K
2	56.7 °C	329.85	K
3	535.70 °C @ 760.00 mmHg (est)	808.85	K
4	57 °C	330.15	K
5	135 °F	330.37222	K

a The blue entry is selected as the recommended value by the software agent. The discrepant entry is colored in red.

#### 3.2.5 Case 5: Navigate TWA Chemistry Domain with Federated Queries

The inherent interoperability of TWA and the according accessibility of other domains and subdomains that are linked to OntoSpecies enable data enrichment. For example, in the OntoCompChem ontology, information on the input and output of DFT calculation are reported. Every conducted computational chemistry calculation (occ:G09) is linked to a species in OntoSpecies by the predicate (occ:hasUniqueSpecies). Information like optimized geometry, HOMO–LUMO gap, and rotational constants can then be accessed through a federated query. A federated query that returns the HOMO–LUMO gaps and the level of theory and basis set of the DFT calculations performed for the carbon dioxide species is reported in Figure S7 in “Supporting Information”.

Four calculations on HOMO–LUMO gaps are stored in OntoCompChem for carbon dioxide, and they are listed in Table 5.

**Table 5:** Level of Theory and Basis Set of the DFT Calculations Performed on the Carbon Dioxide Species and the Calculated HOMO–LUMO Gaps Obtained as the Results of the Federated Query

index	level of theory	basis set	HOMO–LUMO gap [eV]
1	RB3LYP	6-31G(d,p)	–6.0270534
2	RB3LYP	6-31G(d,p)	–10.344957
3	RB3LYP	6-311G(d,p)	–11.055719
4	RB3LYP	6-311+G(d,p)	–9.965902

Other information, like the outputs from a vibrational frequency calculation can be obtained in the same way and processed by an agent to calculate other relevant properties such as heat capacities, entropy, and enthalpy.^61^

### 3.3 Automation of Research Laboratories

The automation of research activities in chemical laboratories plays an important role in accelerating scientific discovery and saving resources. The term “lab automation” is commonly used to describe the development of platforms comprising of robotic handlers that can carry out simple synthesis experiments and analyze results, driven by data-driven algorithms.^90^ These activities aim to realize a “self-driving laboratory”, but fundamental challenges around data availability, human-in-the-loop, and decision-making remain.^91^ We argue, that current approaches are not sufficient to meet these challenges and might even limit further development:^92^ In order to represent and automate all aspects of experimental management, planning, execution, analysis, and reporting, the embedding of deep chemical knowledge (as present in OntoSpecies) is necessary. To achieve this, siloed nature of existing systems has to be overcome,^93^ integrating different data sources and aspects of digitalization such as Lab Inventory Management Systems (LIMS) and Electronic Lab Notebooks (ELN).^7,94^

Organizational and management tasks are critical for smooth operations in a research lab and, most importantly, ensure human safety. For example, the linking of GHS hazard statements to respective chemicals (see “Terminology Component”) is essential for automating chemical hazard identification and risk assessment. Moreover, the availability of big data on chemical structures and associated properties can help in predicting potential products’ characteristics, such as toxicology.^95^ We demonstrated in “Data Access via Complex Queries”, how OntoSpecies can be used in multiobjective experimental planning tasks such as solvent selection.

Accurate representation of experimental steps also requires access to a tacit chemical knowledge. This not only increases experimental repeatability but most importantly enables advanced experimental design algorithms to fully leverage all available data to optimize reaction conditions.^92,96^ In the analytical stage after execution of an experiment, i.e., the actual performance of the laboratory test, OntoSpecies can help in tasks like identification of products by NMR spectra (see “Data Access via Complex Queries”).

In the postanalytical stage, OntoSpecies in combination with other ontology domains of TWA will help sample management and reporting. As an example, sensors have been designed to archive tested samples, reducing errors from mislabeling or incorrect storage. Tracking results, samples, or assets is inherently straightforward as the uniqueness of identifiers (IRIs) is built in by design. Standardized reporting of procedures, observations, and results, which is considered a key challenge,^94,96^ will enabled by the usage of interconnected dynamic KGs. By connecting more and more relevant domain ontologies, we are able to represent and subsequently automate research-related tasks of increasing complexity—working toward the vision of an “AI scientist” that can make Nobel-worthy discoveries, an idea that has been recently introduced in this context.^97^

### 3.4 Implementation Limits

Although OntoSpecies makes the gathering and processing of chemical data easier compared to nonsemantic databases, we identified some weaknesses:

1. To submit a query, the user needs to know the SPARQL language and how the knowledge has been structured in the KG. In some cases of user error, the queries return no data but are considered formally correct, and no warnings are reported. For this reason, a more user-friendly interface with the KG is desired. In TWA context, “Marie”, a question-answering interface that allows the users to type their questions in their natural language, has been developed.^71,72^ However, “Marie” is not acting on the full scope of TWA yet and is currently under further development.
2. The results of the queries reported in the use cases (see “Data Access via Complex Queries” and “Data Enrichment”) depend on the species that have been already instantiated in OntoSpecies. This means that if a species has not been instantiated yet but satisfies the requirements of the query, it will not be shown in the result, leaving out species that can be important in the query context. However, OntoSpecies is automatically growing instantiating many new species every day and those can be selected based on use cases.

## 4 Conclusions

This paper introduces OntoSpecies, a web ontology designed to semantically represent chemical species and their properties. It serves as a core ontology in the TWA chemistry domain. Compared to its previous implementation, it has been extended to include a wide range of identifiers, chemical and physical properties, chemical classifications, and applications, plus spectral information associated with each species, and the provenance and attribution metadata, making it the most comprehensive semantic database on chemical species. The information on a chemical species is collected from the respective PubChem data on the compound using a software agent. In this way, the ontology is enriched with a vast amount of chemical information, resulting in a comprehensive and reliable source of chemical data that can be accessed through a SPARQL end point.

We believe that our approach represents a significant advancement in the field of chemical data management. It offers a standardized way to represent chemical data and provides a powerful means for navigating and analyzing chemical information in a way that is not possible using traditional databases technologies, making it a valuable tool for scientific research. The ontological format permits advanced queries and easy data analysis and visualization. To demonstrate the usefulness of our approach, we presented several use cases that showcase how OntoSpecies can be used to compare chemical properties of similar compounds, find compounds with required characteristics, and automate laborious data gathering by researchers. We show how complex tasks such as the identification of species in an unknown mixture based on NMR measurements, the selection of suitable solvents based on multiple criteria, or the investigation of trends in chemical properties can be addressed using SPARQL queries in combination with the use of software agents to process the information obtained. We also show how the ontological format is beneficial to maintain and enrich the data, as well as to check its consistency and accuracy. Finally, the link between OntoSpecies and other ontologies in TWA is discussed in the context of laboratory automation and cross-domain applications in the TWA ecosystem.

## Supporting Information Available

OntoSpecies ontology (list of classes and their description) and use cases (SPARQL queries and their results) (PDF)
Video to gain insights into the key concepts, methodologies, and findings presented in this work (MP4)

## Notes

Information on how to access OntoSpecies can be found at https://theworldavatar.io/chemistry/documentation/ontologies/ontospecies. The World Avatar knowledge graph and the software agent used to import data from PubChem and populate the knowledge graph are publicly available on GitHub at https://github.com/cambridge-cares/TheWorldAvatar/tree/main/Agents/PubChemAgent under the MIT license. The specific version of the software corresponding to the state at the time of publication can be found in commit “af4024f” within the repository. The CSV file that includes the InChI list used as input to the agent is available in the University of Cambridge data repository (http://doi:10.17863/CAM.97235). OntoSpecies OWL file, OntoSpecies triples in Turtle format as of April 2023, OntoSpecies shape file, OntoSpecies subset of triples in Turtle format used for SHACL validation and the scripts to reproduce the figures and results of all use cases are available in the University of Cambridge data repository (http://doi:10.17863/CAM.97235.2).
