# Supplementary material for: Chemical Species Ontology for Data Integration and Knowledge Discovery
Source: J Chem Inf Model. 2023 Oct 26;63(21):6569–86. doi: 10.1021/acs.jcim.3c00820 (PMC10647085; doi:10.1021/acs.jcim.3c00820)
Supplement: Supplementary file 1 — ci3c00820_si_001.pdf [file ci3c00820_si_001.pdf]

# Supporting Information: A Chemical Species Ontology for Data Integration and Knowledge Discovery

Laura Pascazio,[†] Simon Rihm,[†,‡] Ali Naseri,[‡] Sebastian Mosbach,[†,‡,¶] Jethro Akroyd,[†,‡,¶] and Markus Kraft*,[†,‡,¶,§,‖]

†*CARES, Cambridge Centre for Advanced Research and Education in Singapore, 1 Create Way, CREATE Tower, #05-05, Singapore, 138602*

‡*Department of Chemical Engineering and Biotechnology, University of Cambridge, Philippa Fawcett Drive, Cambridge, CB3 0AS, United Kingdom*

¶*CMCL Innovations, Sheraton House, Castle Park, Cambridge CB3 0AX, United Kingdom*

§*School of Chemical and Biomedical Engineering, Nanyang Technological University, 62 Nanyang Drive, Singapore, 637459*

‖*The Alan Turing Institute, 96 Euston Rd., London, NW1 2DB, United Kingdom*

E-mail: mk306@cam.ac.uk

# Classes in OntoSpecies

A list of all classes in OntoSpecies and their description is reported in Table S1 in alphabetical order. The definitions of namespace prefixes used in Table S1 are summarised in Table 2 in the main text. The table also includes the parent class and the corresponding CHEMINF or CHMO equivalent class when applicable. A class is linked to the parent class and equivalent class through the predicates `rdfs:subClassOf` and `owl:equivalentClass`, respectively.

Table S1: List of classes in OntoSpecies and their description. Parent class and equivalent class are also reported when applicable.

| **Class** | **Description** |
|---|---|
| `os:11BNMRSpectra` | Boron-11 NMR spectroscopy (also known as 11B NMR) is a version of NMR spectroscopy used to elucidate the structure of boron-containing compounds.<br>• Subclass of `os:1DNMRSpectra`<br>• Equivalent to `CHMO:CHMO_0000843` |
| `os:13CNMRSpectra` | Carbon-13 NMR spectroscopy (also known as 13C NMR) is a version of NMR spectroscopy used to elucidate the structure of carbon-containing compounds.<br>• Subclass of `os:1DNMRSpectra`<br>• Equivalent to `CHMO:CHMO_0000837` |
| `os:15NNMRSpectra` | Nitrogen-15 NMR spectroscopy (also known as 15N NMR) is a version of NMR spectroscopy used to elucidate the structure of nitrogen-containing compounds.<br>• Subclass of `os:1DNMRSpectra`<br>• Equivalent to `CHMO:CHMO_0000844` |
| `os:17ONMRSpectra` | Oxygen-17 NMR spectroscopy (also known as 17O NMR) is a version of NMR spectroscopy used to elucidate the structure of oxygen-containing compounds.<br>• Subclass of `os:1DNMRSpectra`<br>• Equivalent to `CHMO:CHMO_0001189` |

| | |
|---|---|
| `os:19FNMRSpectra` | Fluorine-19 NMR spectroscopy (also known as 19F NMR) is a version of NMR spectroscopy used to elucidate the structure of fluorine-containing compounds.<br>• Subclass of `os:1DNMRSpectra`<br>• Equivalent to `CHMO:CHMO_0000845` |
| `os:29SiNMRSpectra` | Silicon-29 NMR spectroscopy (also known as 29Si NMR) is a version of NMR spectroscopy used to elucidate the structure of silicon-containing compounds.<br>• Subclass of `os:1DNMRSpectra`<br>• Equivalent to `CHMO:CHMO_0001955` |
| `os:31PNMRSpectra` | Phosphorus-31 NMR spectroscopy (also known as 31P NMR) is a version of NMR spectroscopy used to elucidate the structure of phosphorus-containing compounds.<br>• Subclass of `os:1DNMRSpectra`<br>• Equivalent to `CHMO:CHMO_0000839` |
| `os:1DNMRSpectra` | One-dimensional NMR spectra.<br>• Subclass of `os:NMRSpectra`<br>• Equivalent to `CHMO:CHMO_0001928` |
| `os:1H13CNMRSpectra` | Two-dimensional 1H-13C NMR spectra.<br>• Subclass of `os:2DNMRSpectra`<br>• Equivalent to `CHMO:CHMO_0000890` |
| `os:1H1HNMRSpectra` | Two-dimensional 1H-1H NMR spectra.<br>• Subclass of `os:2DNMRSpectra`<br>• Equivalent to `CHMO:CHMO_0002420` |
| `os:1HNMRSpectra` | Hydrogen-1 NMR spectroscopy (also known as H1 NMR or proton NMR) is a version of NMR spectroscopy used to elucidate the structure of hydrogen-containing compounds.<br>• Subclass of `os:1DNMRSpectra`<br>• Equivalent to `CHMO:CHMO_0002419` |

| | |
|---|---|
| os:2DNMRSpectra | Two-dimensional NMR spectroscopy is a set of nuclear magnetic resonance spectroscopy (NMR) methods, which give data plotted in a space defined by two frequency axes.<br>• Subclass of os:NMRSpectra<br>• Equivalent to CHMO:CHMO_0000932 |
| gc:Atom | A chemical entity constituting the smallest component of an element having the chemical properties of the element. |
| os:AtomChiralCount | Atom stereocenter (atom that is related to four distinct atoms) count.<br>• Subclass of os:Property<br>• Equivalent to CHEMINF:CHEMINF_000205 |
| os:AtomChiralDefCount | Defined atom stereocenter (atom that is related to four distinct atoms) count.<br>• Subclass of os:Property<br>• Equivalent to CHEMINF:CHEMINF_000206 |
| os:AtomChiralUndefCount | Undefined atom stereocenter (atom that is related to four distinct atoms) count.<br>• Subclass of os:Property<br>• Equivalent to CHEMINF:CHEMINF_000212 |
| os:AtomicBond | Bond between two atoms.<br>• Equivalent to CHEMINF:CHEMINF_000063 |
| os:AtomicRadius | Radius of an atom.<br>• Subclass of os:Property<br>• Equivalent to CHEMINF:CHEMINF_000125 |
| os:AtomicWeight | Mass of an atom.<br>• Subclass of os:Property<br>• Equivalent to CHEMINF:CHEMINF_000084 |

| | |
|---|---|
| os:AutoignitionTemperature | The lowest temperature at which the substance will spontaneously ignite in a normal atmosphere without an external source of ignition (e.g., spark or flame).<br>• Subclass of os:Property<br>• Equivalent to CHEMINF:CHEMINF_000444 |
| os:BoilingPoint | The temperature at which this compound changes state from liquid to gas at a given atmospheric pressure.<br>• Subclass of os:ThermoProperty<br>• Equivalent to CHEMINF:CHEMINF_000257 |
| os:BondChiralCount | Bond stereocenter count.<br>• Subclass of os:Property<br>• Equivalent to CHEMINF:CHEMINF_000213 |
| os:BondChiralDefCount | Defined bond stereocenter count.<br>• Subclass of os:Property<br>• Equivalent to CHEMINF:CHEMINF_000214 |
| os:BondChiralUndefCount | Undefined bond stereocenter count.<br>• Subclass of os:Property<br>• Equivalent to CHEMINF:CHEMINF_000215 |
| os:Caco2Permeability | Caco-2 (Cancer coli-2) is a human colon epithelial cancer cell line (established from human colorectal adenocarcinoma cells). It is primarily used as a model of the intestinal epithelial barrier. The Caco-2 permeability of a chemical is used as a measure of its intestinal absorption in human.<br>• Subclass of os:Property |
| os:CanonicalizedCompound | Indicate if the compound is canonicalized.<br>• Subclass of os:Property |
| os:Charge | Total charge of a chemical entity.<br>• Subclass of os:Property<br>• Equivalent to CHEMINF:CHEMINF_000131 |

| | |
|---|---|
| os:ChebiID | Database identifier used by ChEBI.<br>• Subclass of os:Identifier<br>• Equivalent to CHEMINF:CHEMINF_000407 |
| os:ChemicalClass | Chemical classes are groupings that relate chemicals by similar features. Chemicals can be classified by their structure (e.g., hydrocarbons), uses (e.g., pesticides), physical properties (e.g., volatile organic compounds [VOCs]), radiological properties (e.g., radioactive materials), or other factors. ChEBI Ontology tree. ChEBI is an acronym for Chemical Entities of Biological Interest, which is a freely available dictionary of molecular entities focused on 'small' chemical compounds. ChEBI incorporates an ontological classification, whereby the relationships between molecular entities or classes of entities and their parents and/or children are specified.<br>• Subclass of os:Classification |
| os:CID | Database identifier used by PubChem.<br>• Subclass of os:Identifier<br>• Equivalent to CHEMINF:CHEMINF_000140 |
| os:Classification | A set of concepts and categories in a subject area or domain that shows their properties and the relations between them. |
| os:CollisionCrossSection | Collision cross section represents the effective area for the interaction between an individual ion and the neutral gas through which it is traveling (e.g., in ion mobility spectrometry experiments). It quantifies the probability of a collision taking place between two or more particles.<br>• Subclass of os:Property |
| os:CompoundComplexity | Indicator that denotes how complicated a structure is.<br>• Subclass of os:Property<br>• Equivalent to CHEMINF:CHEMINF_000390 |

| | |
|---|---|
| `os:CovalentUnitCount` | The number of covalent units in a chemical structure.<br>• Subclass of `os:Property`<br>• Equivalent to `CHEMINF:CHEMINF_000280` |
| `os:Density` | Density (with unit) and specific gravity (without unit) of a compound. Density is mass of a unit volume of a compound and commonly expressed in units of kg/m3 or g/cm3. Specific gravity, also known as relative density, is a unit-less quantity, defined as the ratio of the density of a compound to that of a standard reference material (typically, water at 4 °C for liquids and air at room temperature [20 °C or 68 °F] for gases).<br>• Subclass of `os:ThermoProperty`<br>• Equivalent to `CHEMINF:CHEMINF_000416` |
| `os:DissociationConstants` | A specific type of equilibrium constant that measures the propensity of a larger object to separate (dissociate) reversibly into smaller components, as when a complex falls apart into its component molecules, or when a salt splits up into its component ions. This includes pKa (the negative logarithm of the acid dissociation constant) and pKb (the negative logarithm of the base dissociation constant).<br>• Subclass of `os:ThermoProperty` |
| `os:ElectronAffinity` | Amount of energy released when an electron attaches to a neutral atom or molecule in the gaseous state to form an anion.<br>• Subclass of `os:Property` |
| `os:ElectronConfiguration` | Arrangement of electrons in orbitals around an atomic nucleus.<br>• Subclass of `os:Property` |

| | |
|---|---|
| os:Electronegativity | Electronegativity is an atomic quality that describes its power to attract electrons to itself.<br>• Subclass of os:Property<br>• Equivalent to CHEMINF:CHEMINF_000121 |
| pt:Element | An element in the periodic table. |
| os:ElementClassification | Classification of elements in the periodic table.<br>• Subclass of os:Classification |
| os:ElementGroupNumber | Group number of an element in the periodic table.<br>• Subclass of os:Property |
| os:ElementName | Name of an element in the periodic table.<br>• Subclass of os:Identifier |
| os:ElementPeriodNumber | Period number of an element in the periodic table.<br>• Subclass of os:Property |
| os:ElementSymbol | Symbol of an element in the periodic table.<br>• Subclass of os:Identifier |
| os:EnthalpyOfSublimation | The enthalpy (or heat) of sublimation is the amount of energy that must be added to a mole of solid at constant pressure to turn it directly into a gas (without passing through the liquid phase).<br>• Subclass of os:ThermoProperty |
| os:ExactMass | Mass of the most intense molecule peak in an MS spec, and when calculated denotes the mass of a molecule containing most likely isotopic composition for a single random molecule<br>• Subclass of os:Property<br>• Equivalent to CHEMINF:CHEMINF_000217 |
| os:FlashPoint | The lowest temperature at which a liquid can gives off vapor to form an ignitable mixture in air near the surface of the liquid.<br>• Subclass of os:ThermoProperty<br>• Equivalent to CHEMINF:CHEMINF_000417 |

| | |
|---|---|
| os:Frequency | Frequency of the spectrometer. |
| os:FunctionalGroup | Specific groups of atoms within molecules that are responsible for the characteristic chemical reactions of those molecules.<br>• Equivalent to CHEMINF:CHEMINF_000068 |
| os:GCMS | Data from gas chromatography-mass spectrometry (GC-MS) experiments.<br>• Subclass of os:MassSpectrometry<br>• Equivalent to CHMO:CHMO_0000497 |
| os:Geometry | Geometry of a molecule. |
| os:GHSHazardStatement | GHS (Globally Harmonized System of Classification and Labelling of Chemicals) is a United Nations system to identify hazardous chemicals and to inform users about these hazards. GHS has been adopted by many countries around the world and is now also used as the basis for international and national transport regulations for dangerous goods.<br>• Subclass of os:Classification |
| os:GroundLevel | Ground level of an element in the periodic table.<br>• Subclass of os:Property |
| os:HeatOfCombustion | The heat of combustion is the energy released as heat when a compound undergoes complete combustion with oxygen under standard conditions.<br>• Subclass of os:ThermoProperty |
| os:HeatOfVaporization | The heat (or enthalpy) of vaporization is the quantity of heat that must be absorbed if a certain quantity of liquid is vaporized at a constant temperature.<br>• Subclass of os:ThermoProperty<br>• Equivalent to CHEMINF:CHEMINF_000418 |

| | |
|---|---|
| os:HeavyAtomCount | The number of non-hydrogen atoms.<br>• Subclass of os:Property<br>• Equivalent to CHEMINF:CHEMINF_000300 |
| os:HenrysLawConstant | Henry's law states that the amount of dissolved gas (in liquid, such as water) is proportional to its partial pressure in the gas phase. The proportionality factor is called the Henry's law constant and defined as the ratio of a compound's partial pressure in air to the concentration of the compound in water at a given temperature.<br>• Subclass of os:ThermoProperty<br>• Equivalent to CHEMINF:CHEMINF_000433 |
| os:HydrogenBondAcceptorCount | Number of hydrogen bond acceptors in a given molecular entity. This is usually the count of all negatively or partially negatively charged heteroatoms (e.g. alcohol oxygen) capable of accepting a hydrogen bond.<br>• Subclass of os:Property<br>• Equivalent to CHEMINF:CHEMINF_000245 |
| os:HydrogenBondDonorCount | Number of hydrogen bond donors in a given molecular entity. This is usually the count of all negatively or partially negatively charged heteroatoms (e.g. alcohol oxygen) that have covalently attached to them partially positively charged hydrogen atoms that are capable of participating in a hydrogen bond.<br>• Subclass of os:Property<br>• Equivalent to CHEMINF:CHEMINF_000244 |
| os:Hydrophobicity | Hydrophobicity is the physical property of a molecule that is seemingly repelled from a mass of water.<br>• Subclass of os:Property |
| os:Identifier | Chemical names, synonyms, identifiers, and descriptors.<br>• Equivalent to CHEMINF:CHEMINF_000061 |

| | |
|---|---|
| `os:InChI` | The IUPAC International Chemical Identifier (InChI) is a textual identifier for chemical substances, designed to provide a standard and human-readable way to encode molecular information and to facilitate the search for such information in databases and on the web.<br>• Subclass of `os:Identifier`<br>• Equivalent to `CHEMINF:CHEMINF_000113` |
| `os:InChIKey` | The InChIKey is a fixed length (27 character) condensed digital representation of the InChI that is not human-understandable.<br>• Subclass of `os:Identifier`<br>• Equivalent to `CHEMINF:CHEMINF_000059` |
| `os:InstrumentType` | Type of instrument used for the spectrometry. |
| `os:IonizationMode` | Ionization mode used in the mass spectrometry analysis. |
| `os:IonizationPotential` | Ionization potential, also called ionization energy, is the amount of energy required to remove an electron from an isolated atom or molecule.<br>• Subclass of `os:Property`<br>• Equivalent to `CHEMINF:CHEMINF_000191` |
| `os:IsoelectricPoint` | The isoelectric point, sometimes abbreviated to IEP, is the pH at which a particular molecule or surface carries no net electrical charge.<br>• Subclass of `os:Property` |
| `os:IsotopeAtomCount` | The sum of all atoms enriched with respect to a particular atom isotope.<br>• Subclass of `os:Property`<br>• Equivalent to `CHEMINF:CHEMINF_000301` |

| | |
|---|---|
| os:IUPACName | An IUPAC name is a systematic name which is formulated according to the rules and recommendations for chemical nomenclature set out by the International Union of Pure and Applied Chemistry (IUPAC).<br>• Subclass of os:Identifier<br>• Equivalent to CHEMINF:CHEMINF_000107 |
| os:LCMS | Data from liquid chromatography-mass spectrometry (LC-MS) experiments.<br>• Subclass of os:MassSpectrometry<br>• Equivalent to CHMO:CHMO_0000524 |
| os:LogP | Log P is the partition coefficient expressed in logarithmic form. The partition coefficient is the ratio of concentrations of a compound in a mixture of two immiscible solvents at equilibrium. This ratio is therefore used to compare the solubilities of the solute in these two solvents. Because octanol and water are the most commonly used pair of solvents for measuring partition coefficients, the Log P values listed in this section refer to octanol/water partition coefficients, unless indicated otherwise.<br>• Subclass of os:Property<br>• Equivalent to CHEMINF:CHEMINF_000251 |
| os:LogS | The base-10 logarithm of the aqueous solubility of this compound.<br>• Subclass of os:Property |
| os:MALDI | MALDI (matrix-assisted laser desorption/ionization) is an ionization technique that uses a laser energy absorbing matrix to create ions from large molecules with minimal fragmentation.<br>• Subclass of os:MassSpectrometry<br>• Equivalent to CHMO:CHMO_0002203 |

| | |
|---|---|
| os:MSMS | Data from tandem mass spectrometry (MS-MS) experiments.<br>• Subclass of os:MassSpectrometry<br>• Equivalent to CHMO:CHMO_0000701 |
| os:MassSpectrometry | Mass spectrometry (MS or mass spec) is a technique to determine molecular structure through ionization and fragmentation of the parent compound into smaller components.<br>• Subclass of os:SpectralInformation<br>• Equivalent to CHMO:CHMO_0000470 |
| os:MeltingPoint | The melting point is the temperature at which a substance changes state from solid to liquid at atmospheric pressure. When considered as the temperature of the reverse change (from liquid to solid), it is referred to as the freezing point.<br>• Subclass of os:ThermoProperty<br>• Equivalent to CHEMINF:CHEMINF_000256 |
| os:MolecularFormula | A molecular formula is a structure descriptor which identifies each constituent element by its chemical symbol and indicates the number of atoms of each element found in each discrete molecule of that compound.<br>• Subclass of os:Identifier<br>• Equivalent to CHEMINF:CHEMINF_000042 |
| os:MolecularWeight | Mass of a molecule.<br>• Subclass of os:Property<br>• Equivalent to CHEMINF:CHEMINF_000216 |
| os:MonoIsotopicWeight | The mass of a molecule calculated using the mass of the most abundant isotope of each element (e.g., Carbon has a monoisotopic mass of 12.000 g/mol).<br>• Subclass of os:Property<br>• Equivalent to CHEMINF:CHEMINF_000218 |

| | |
|---|---|
| os:NMRSpectra | Nuclear magnetic resonance spectrum.<br>• Subclass of os:SpectralInformation<br>• Equivalent to CHMO:CHMO_0000835 |
| os:OpticalRotation | Optical rotation is a property of chiral substances that is expressed as the angle to which the material causes polarized light to rotate at a particular temperature, wavelength, and concentration.<br>• Subclass of os:ThermoProperty<br>• Equivalent to CHMO:CHMO_0002818 |
| os:OtherMS | Other compound's mass spectrometry (MS) information.<br>• Subclass of os:MassSpectrometry |
| os:OxidationStates | Oxidation states of an element in the periodic table.<br>• Subclass of os:Property |
| os:Peak | A peak of the spectrum. |
| os:PolarSurfaceArea | The polar surface area is defined as the combined surface area belonging to oxygen and nitrogen atoms and hydrogen atoms bound to these electronegative atoms.<br>• Subclass of os:Property<br>• Equivalent to CHEMINF:CHEMINF_000307 |
| os:Property | Chemical or physical property of a chemical species or element in the periodic table. |
| okin:Reference | Provenance of data. |
| os:ReferenceState | Reference state of a thermodynamic property. |
| os:RotatableBondCount | A bond count that denotes the integer number of rotors in the molecule, generally single bonds torsion around which produces non-identical geometric molecular configurations.<br>• Subclass of os:Property<br>• Equivalent to CHEMINF:CHEMINF_000254 |

| | |
|---|---|
| os:SMILES | A SMILES is a structure descriptor that denotes a molecular structure as a graph.<br>• Subclass of os:Identifier<br>• Equivalent to CHEMINF:CHEMINF_000018 |
| os:Solubility | The solubility of a substance is the amount of that substance that will dissolve in a given amount of solvent. The default solvent is water, if not indicated.<br>• Subclass of os:ThermoProperty<br>• Equivalent to CHEMINF:CHEMINF_000258 |
| os:Solvent | Solvent used in the NMR analysis. |
| os:Species | An ensemble of chemically identical molecular entities.[S1] |
| os:SpectraGraph | Spectral data collected in a graph. |
| os:SpectralInformation | Spectral data for this compound, including 1-D and 2-D NMR, Infrared (IR), Raman, and Ultraviolet (UV) spectroscopy, mass spectrometry (MS), and chromatography.<br>• Equivalent to CHMO:CHMO_0000800 |
| os:StandardEnthalpyOfFormation | The energy required to form 1 mole of a substance from its constituent elements.<br>• Subclass of os:ThermoProperty<br>• Equivalent to CHEMINF:CHEMINF_000241 |
| os:SubStructureKeysFingerprint | Binary substructure fingerprint for chemical structures. These fingerprints are used for similarity neighboring and similarity searching.<br>• Subclass of os:Property |
| os:SurfaceTension | Surface tension is a contractive tendency of the surface of a liquid that allows it to resist an external force. It is measured as the energy required to increase the surface area of a liquid by a unit of area.<br>• Subclass of os:ThermoProperty<br>• Equivalent to CHEMINF:CHEMINF_000420 |

| | |
|---|---|
| os:TautomerCount | Count of tautomers.<br>• Subclass of os:Property<br>• Equivalent to CHEMINF:CHEMINF_000202 |
| os:ThermoProperty | Thermodynamic property of a chemical species.<br>• Subclass of os:Property |
| om:Unit | Unit of measurement. |
| os:Use | Application and role of a chemical species. |
| os:VaporDensity | The density of a gas or vapor relative to that of the reference gas. While some resources use the hydrogen gas as the reference gas for the vapor density calculation, many resources (particularly in relation to safety considerations at commercial and industrial facilities in the U.S.) defines the vapor density with respect to the density of air, which has an arbitrary value of one. If a gas has a vapor density of less than one it will generally rise in air. If the vapor density is greater than one the gas will generally sink in air.<br>• Subclass of os:ThermoProperty<br>• Equivalent to CHEMINF:CHEMINF_000440 |
| os:VaporPressure | Vapor pressure (or equilibrium vapor pressure) is the pressure of a vapor in thermodynamic equilibrium with its condensed phases in a closed system.<br>• Subclass of os:ThermoProperty<br>• Equivalent to CHEMINF:CHEMINF_000419 |
| os:Viscosity | Viscosity is a measure of a fluid's resistance to flow. It describes the internal friction of a moving fluid.<br>• Subclass of os:ThermoProperty |
| os:XCoordinate | Atom X coordinate in space. |

| | |
|---|---|
| `os:XLogP3` | XLOGP3is an atom-additive method that calculates log P by adding up contributions from each atom in the given molecule.<br>• Subclass of `os:Property` |
| `os:YCoordinate` | Atom Y coordinate in space. |
| `os:ZCoordinate` | Atom Z coordinate in space. |

# Data Validation Using SHACL

To ensure the structural and semantic integrity of OntoSpecies ABoxes, we employed the Shapes Constraint Language (SHACL) for validation.[S2] SHACL is a powerful language for expressing constraints on RDF data and is recommended by the W3C for RDF validation. TBox concepts from our ontology have been translated into SHACL shapes, providing a precise representation of the expected structure and semantics for our RDF data validation. These shapes define constraints such as properties, their range and domain, datatypes, and other structural requirements. After the data import, a subset of ABoxes, comprising the triples associated with 500 species (randomly selected from the larger dataset of ≈36,000 species, as of April 2023) was validated. Validating the entire dataset of approximately 46 million triples was impractical due to its scale. Nevertheless, this validation process provides a rigorous and comprehensive examination, ensuring that the RDF data aligns precisely with the anticipated structure and semantics. Any disparities or inconsistencies detected during this procedure were meticulously identified, prompting a thorough review and resolution to uphold the highest levels of data integrity. This validation approach serves as a robust 'unit test' for our data import process, assuring not only accurate data importation but also strict adherence to structural and semantic standards. Detailed information on how to access the SHACL shape file and the validation data subset can be found in the 'Data and Software Availability' section in the main text.

# Data Access via Complex Queries

## USE CASE 1: Alkanes Ionization Energy Trend

The size-dependence of alkanes' ionisation energies is still a topic of ongoing research.[S3] The ionization energy of a species is represented within OntoSpecies. Figure S1 shows that the trend of the experimental ionization energies of hydrocarbons observed and used to develop an underlying model by Bakulin et al.[S3] was qualitatively reproduced by data queried via OntoSpecies (available via PubChem).

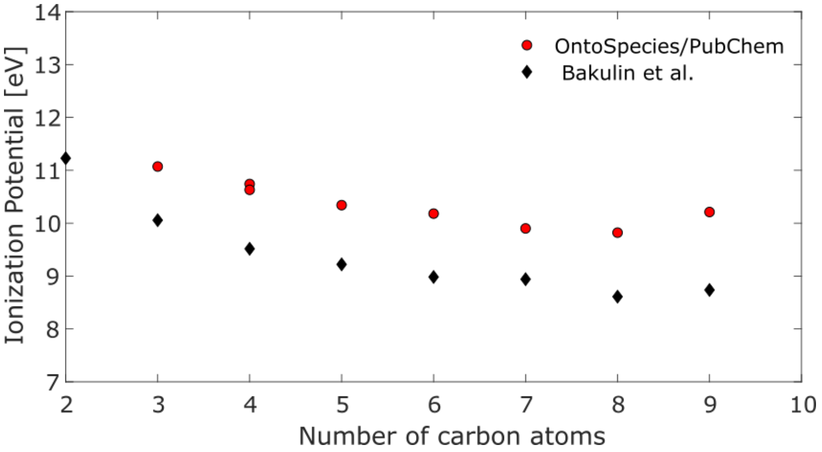


Figure S1: Comparison between the experimental trends of ionisation energies of saturated hydrocarbons with increasing carbon chain length taken from OntoSpecies/PubChem (red circles) and Bakulin et al. (black diamonds).

## USE CASE 2: SPARQL Query

Figure S2 shows the SPARQL query that selects all species that can be used as co-solvents for propan-2-ol and are classified as alcohols. We decided to use alcohols because they are generally more desirable than solvents in other classes (e.g. bases or ethers class).[S4]

Three selection criteria are included in the SPARQL query:

- CRITERION 1: The co-solvent boiling point needs to be 15 K lower or higher than the propan-2-ol boiling point to ensure ease of separation between the two solvents by distillation (355 K).

```
PREFIX os: <http://www.theworldavatar.com/ontology/ontospecies/OntoSpecies.owl#>
PREFIX rdf: <http://www.w3.org/1999/02/22-rdf-syntax-ns#>
PREFIX rdfs: <http://www.w3.org/2000/01/rdf-schema#>

SELECT DISTINCT ?speciesIRI ?iupacstring
WHERE {
   # select a species and its IUPAC name
   # ?iupac - species IUPAC name IRI
   # the filter on the IUPAC name IRI selects the IUPAC name indexed 1
   ?speciesIRI rdf:type os:Species ; rdfs:label ?formula ; os:hasIUPACName ?iupac .
   ?iupac os:value ?iupacstring .
   FILTER(regex(str(?iupac) ,'_1_'))

   # select species classified as "alcohol" navigating the classification tree
   ?speciesIRI (rdf:|!rdf:)* ?x .
   ?x ?y ?z .
   ?z (rdf:|!rdf:)* ?classIRI .
   ?classIRI rdfs:label "alcohol" .
   FILTER( regex(?formula, "C[0-9]{0,3}H[0-9]{0,3}O[0-9]{0,3}$") )

   # select species used as "solvent"
   ?speciesIRI os:hasUse ?use .
   ?use rdfs:label ?usestring .
   FILTER(regex(lcase(?usestring), "solvent"))

   ########################################################################################
   #      COMMENT/UNCOMMENT code blocks to use a different combination of criteria        #
   ########################################################################################

   ##################################### CRITERION 1 ######################################
   # select species with boiling point ?BPValue < Tb - 15 K and ?BPValue > Tb + 15 K
   # where Tb = 355 K (propan-2-ol boiling point)
   ?speciesIRI os:hasBoilingPoint ?BP .
   ?BP os:value ?BPValue ; os:unit ?u .
   ?u rdfs:label "K" .
   FILTER(?BPValue > 370 || ?BPValue < 340)

   ##################################### CRITERION 3 ######################################
   # select species with boiling point lower than 423 K
   FILTER(?BPValue < 423)

   ##################################### CRITERION 2 ######################################
   # select species that do not have hazard statements related to unborn child or cancer
   FILTER NOT EXISTS{
      ?speciesIRI os:hasGHSHazardStatements ?ghs .
      ?ghs rdfs:comment ?ghsstring ; rdfs:label ?ghscode .
      FILTER((regex(?ghsstring,'unborn child') || regex(?ghsstring,'cancer'))  && regex(?ghscode,'H3'))
   }
} ORDER BY ?iupacstring
```

Figure S2: SPARQL query that identify suitable co-solvents for propan-2-ol that enable easiest separation by distillation (CRITERION 1), reduce potential health impact (CRITERION 2) and is cost-effective for solvent recovery (CRITERION 3).

- CRITERION 2: Exclude co-solvents with high potential health impact. To do so, we removed all the species that have GHS safety statement related to risk of cancer and risk for unborn child.[S5]

- CRITERION 3: Exclude co-solvents with a boiling point higher than 423 K to reduce

the cost of solvent recovery by distillation.[S4]

## USE CASE 2: List of Suitable Co-Solvents

Table S2 reports a list of co-solvents for propan-2-ol to enable the easiest separation by distillation obtained querying OntoSpecies. First column reports a list of species selected by the SPARQL query in Figure S2 as suitable co-solvents (49 species). The second column reports a check-mark if the species follow criterion 1; the third column reports a check-mark if the species follow criteria 1 and 2 together; the firth column reports a check-mark if the species follow criteria 1, 2 and 3 together. Species that follow criterion 1 but are discarded by criterion 2 are highlighted in red (4 species). Species that follow all the criteria are highlighted in green (13 species).

Table S2: List of co-solvents for propan-2-ol to enable the easiest separation by distillation

| **Species** | **Criterion 1** | **Criteria 1-2** | **Criteria 1-2-3** |
|---|---|---|---|
| 2,2-bis(hydroxymethyl)propane-1,3-diol | ✓ | ✓ | × |
| 2,6-dimethylheptan-4-ol | ✓ | ✓ | × |
| 2-(2-butoxyethoxy)ethanol | ✓ [S4] | ✓ | × [S4] |
| 2-(2-ethoxyethoxy)ethanol | ✓ | ✓ | × |
| 2-(2-hydroxyethoxy)ethanol | ✓ | ✓ | × |
| 2-(2-methoxyethoxy)ethanol | ✓ | × | - |
| 2-(4-methylcyclohex-3-en-1-yl)propan-2-ol | ✓ | ✓ | × |
| 2-[2-(2-hydroxyethoxy)ethoxy]ethanol | ✓ | ✓ | × |
| 2-[2-(2-methoxyethoxy)ethoxy]ethanol | ✓ | ✓ | × |
| 2-[2-[2-(2-hydroxyethoxy)ethoxy]ethoxy]ethanol | ✓ | ✓ | × |
| 2-butoxyethanol | ✓ | ✓ | × |
| 2-butyloctan-1-ol | ✓ | ✓ | ✓ |
| 2-ethoxyethanol | ✓ | × | - |
| 2-ethyl-2-(hydroxymethyl)propane-1,3-diol | ✓ | ✓ | × |
| 2-ethylhexan-1-ol | ✓ | ✓ | × |
| 2-furylmethanol | ✓ | × | - |
| 2-hydroxyacetic acid | ✓ | ✓ | ✓ |
| 2-methoxyethanol | ✓ [S4] | × [S5] | - |
| 2-methylbutan-1-ol | ✓ | ✓ | ✓ |
| 2-methylbutan-2-ol | ✓ | ✓ | ✓ |
| 2-methylpentane-2,4-diol | ✓ | ✓ | × |
| 2-methylpropan-1-ol | ✓ | ✓ | ✓ |
| 2-phenylpropan-2-ol | ✓ | ✓ | × |
| 3-methylbutan-2-ol | ✓ | ✓ | ✓ |

| | | | |
|---|---|---|---|
| butan-1-ol | ✓ [S4] | ✓ | ✓ [S4] |
| butan-2-ol | ✓ | ✓ | ✓ |
| butane-1,2-diol | ✓ | ✓ | × |
| butane-1,3-diol | ✓ | ✓ | × |
| butane-1,4-diol | ✓ | ✓ | × |
| cyclohexanol | ✓ | ✓ | × |
| decan-1-ol | ✓ | ✓ | ✓ |
| dodecan-1-ol | ✓ | ✓ | × |
| ethyl 2-hydroxypropanoate | ✓ | ✓ | × |
| ethylene glycol | ✓ [S4] | ✓ | × [S4] |
| glycerol | ✓ | ✓ | × |
| hexadecan-1-ol | ✓ | ✓ | × |
| hexan-1-ol | ✓ | ✓ | × |
| hexane-1,6-diol | ✓ | ✓ | × |
| icosan-1-ol | ✓ | ✓ | × |
| methanol | ✓ [S4] | ✓ | ✓ [S4] |
| octadecan-1-ol | ✓ | ✓ | × |
| octan-2-ol | ✓ | ✓ | × |
| pentan-1-ol | ✓ | ✓ | ✓ |
| pentan-2-ol | ✓ | ✓ | ✓ |
| pentane-1,5-diol | ✓ | ✓ | × |
| phenylmethanol | ✓ | ✓ | × |
| propan-1-ol | ✓ | ✓ | ✓ |
| propane-1,2-diol | ✓ | ✓ | × |
| propane-1,3-diol | ✓ | ✓ | × |

## USE CASE 3: SPARQL Queries

The SPARQL query that selects all the possible liquid products of the electrochemical $CO_2$ reduction[S6] is shown in Figure S3. The query selects species with chemical formula $C_xH_yO_z$ and with $x < 5$ and $z < 10$. An additional filter on the boiling point ($T_b$) is added to remove all the species that are not expected to be found in the liquid phase at room temperature. The selected $T_b = 15\,°C$ is lower than then the experimental temperature ($T = 25\,°C$) as to ensure all species that might partially be in the liquid phase are included.

The IRI of each species is then used in a second query (Figure S4) to get the $^1$H NMR spectral information (peaks shift and intensity). A filter is used in the query to select only the first $^1$H NMR spectra added to OntoSpecies from the PubChem record. This is done through the IRI indexing described in the "Assertion Component" section in the main text.

```
PREFIX os: <http://www.theworldavatar.com/ontology/ontospecies/OntoSpecies.owl#>
PREFIX rdf: <http://www.w3.org/1999/02/22-rdf-syntax-ns#>
PREFIX rdfs: <http://www.w3.org/2000/01/rdf-schema#>

SELECT DISTINCT ?speciesIRI ?formula
WHERE {
   # select a species and its IUPAC name
   # ?formula - species chemical formula
   ?speciesIRI rdf:type os:Species ; rdfs:label ?formula ;
   FILTER( regex(?formula, "C[0-5]{0,1}H[0-9]{0,2}O[0-9]{0,2}$") )

   # select species with boiling point higher than 288 K
   ?speciesIRI os:hasBoilingPoint ?BP .
   ?BP os:value ?BPValue ; os:unit ?u .
   ?u rdfs:label "K" .
   FILTER(?BPValue > 288)
}
```

Figure S3: SPARQL query selecting all the possible products of the electrochemical reaction expected to be found in the catholyte.

```
PREFIX os: <http://www.theworldavatar.com/ontology/ontospecies/OntoSpecies.owl#>
PREFIX rdf: <http://www.w3.org/1999/02/22-rdf-syntax-ns#>

SELECT ?spectra ?chemicalshift ?intensity
WHERE {
   # select the chemical shift and intensity of each peach of the 1H NMR spectra
   # <#IRI#> in the query text is replaced with the species IRI
   <#IRI#> os:has1HNMRSpectra ?spectra .
   ?spectra os:hasSpectraGRaph ?graph .
   ?graph os:hasPeak ?peak .
   ?peak os:hasX1 ?chemicalshift ; os:hasY ?intensity .
   FILTER( regex(str(?spectra), "_1_") ) # the filter selects only the 1H NMR spectra with index 1
}
```

Figure S4: SPARQL query selecting $^{1}$H NMR spectra peaks information (chemical shift and intensity) of a specific species. #IRI# in the shown query text is replaced with the species IRI.

## USE CASE 3: Subproducts Identification

In the NMR spectra in Figure 8 (main text), the triplet signals at 1.04 ppm do not exactly follow the expected 1:2:1 pattern (i.e. 25%:50%:25%). Calculated peak ares give 30%:42%:27%, which indicates that another signal might overlap on the left side of the triplet in Figure 8 (main text). Also the signals of the quartet at 3.51 ppm do not exactly follow the expected 1:3:3:1 pattern (i.e. 13%:37%:37%:13%). Calculated peak ares give 12%:33%:38%:17%, which indicates that another signal might overlap on the right side of the quartet in Figure 8 (main text). A list of possible subproducts can be then found querying for species whose

highest peak can interfere with these two peaks ($P_3$ and $P_6$). The SPARQL query result is reported in Table S3. Check-marks in columns $P_3$ and $P_6$ indicate that the species interferes with $P_3$ and $P_6$ respectively. Species that interfere with both peaks are highlighted in blue.

Table S3: List of possible subproducts for the NMR spectra in Figure 8 (main text).

| **Species** | $P_3$ | $P_6$ |
|---|---|---|
| (2S,3R)-butane-1,2,3,4-tetrol | ✓ | × |
| (2S,4R)-pentane-1,2,3,4,5-pentol | ✓ | × |
| 2-ethoxyethanol | ✓ | ✓ |
| 2-methoxy-2-methyl-propane | × | ✓ |
| 2-methylbutan-2-ol | × | ✓ |
| 2-methylbutanal | × | ✓ |
| 2-methylbutanoic acid | × | ✓ |
| 2-methylpropan-1-ol | ✓ | ✓ |
| 2-methylpropanal | × | ✓ |
| 2-methylpropanoic acid | × | ✓ |
| 3-methyl-2-oxo-butanoic acid | × | ✓ |
| 3-methylbutan-2-ol | × | ✓ |
| 3-methylbutanoic acid | × | ✓ |
| butan-1-ol | ✓ | ✓ |
| butan-2-ol | ✓ | ✓ |
| butanal | × | ✓ |
| butane-1,3-diol | ✓ | ✓ |
| butane-2,3-diol | ✓ | ✓ |
| butyric acid | × | ✓ |
| ethyl propanoate | × | ✓ |
| ethylene glycol | ✓ | × |
| glycerol | ✓ | × |
| methanol | ✓ | × |
| methyl 2-methylpropanoate | ✓ | ✓ |
| methyl 3-oxobutanoate | ✓ | × |
| methyl acetate | ✓ | × |
| pent-1-en-3-ol | × | ✓ |
| pent-1-en-3-one | × | ✓ |
| pentan-1-ol | ✓ | ✓ |
| pentan-2-ol | × | ✓ |
| pentanal | × | ✓ |
| propan-1-ol | ✓ | ✓ |
| propan-2-ol | × | ✓ |
| propionic acid | × | ✓ |
| tetrahydrofuran | ✓ | × |

# Data Enrichment

## CASE 1: List of Alkenes Boiling Points

Table S4: List of species classified as alkenes in OntoSpecies ordered by number of carbon atoms, their experimental boiling points ($T_{b-experimental}$) taken from PubChem or GuideChem (black or blue color respectively) and their extrapolated boiling points obtained fitting PubChem data with a cube root function ($T_{b-predicted}$).

| **Species** | | $T_{b-experimental}$ [K] | $T_{b-predicted}$ **[K]** |
|---|---|---|---|
| ethylene | $C_2H_4$ | 169.43 | 166.57 |
| prop-1-ene | $C_3H_6$ | 225.43 | 223.04 |
| 2-methylprop-1-ene | $C_4H_8$ | 266.26 | 267.99 |
| but-1-ene | $C_4H_8$ | 267.04 | 267.99 |
| 2-methylbut-1-ene | $C_5H_{10}$ | 304.35 | 305.94 |
| 2-methylbut-2-ene | $C_5H_{10}$ | 310.65 | 305.94 |
| 3-methylbut-1-ene | $C_5H_{10}$ | 293.25 | 305.94 |
| (E)-hex-3-ene | $C_6H_{12}$ | 338.75 | 339.12 |
| 2-methylpent-1-ene | $C_6H_{12}$ | 335.26 | 339.12 |
| hex-1-ene | $C_6H_{12}$ | 336.65 | 339.12 |
| (E)-oct-2-ene | $C_8H_{16}$ | 398.55 | 395.76 |
| (E)-oct-3-ene | $C_8H_{16}$ | 394.05 | 395.76 |
| 3-methyleneheptane | $C_8H_{16}$ | 390.85 | 395.76 |
| 4,5-dimethylhex-1-ene | $C_8H_{16}$ | 380.05 | 395.76 |
| oct-1-ene | $C_8H_{16}$ | 394.43 | 395.76 |
| (E)-2,6-dimethyloct-3-ene | $C_{10}H_{20}$ | - | 443.58 |
| dec-1-ene | $C_{10}H_{20}$ | 443.76 | 443.58 |
| (E)-7-methyldec-4-ene | $C_{11}H_{22}$ | - | 465.12 |
| 5-methyldec-1-ene | $C_{11}H_{22}$ | 456.85 | 465.12 |
| undec-1-ene | $C_{11}H_{22}$ | 465.87 | 465.12 |
| (E)-dodec-2-ene | $C_{12}H_{24}$ | - | 485.38 |
| (E)-dodec-3-ene | $C_{12}H_{24}$ | - | 485.38 |
| 8-methylundec-1-ene | $C_{12}H_{24}$ | 478.15 | 485.38 |
| dodec-1-ene | $C_{12}H_{24}$ | 485.93 | 485.38 |
| (E)-tridec-2-ene | $C_{13}H_{26}$ | 508.45 | 504.56 |
| tetradec-1-ene | $C_{14}H_{28}$ | 524.26 | 522.77 |
| pentadec-1-ene | $C_{15}H_{30}$ | 541.65 | 540.13 |
| hexadec-1-ene | $C_{16}H_{32}$ | 557.55 | 556.74 |
| heptadec-1-ene | $C_{17}H_{34}$ | - | 572.67 |
| (E)-octadec-7-ene | $C_{18}H_{36}$ | 595.35 | 587.98 |
| (E)-octadec-9-ene | $C_{18}H_{36}$ | 595.35 | 587.98 |
| octadec-1-ene | $C_{18}H_{36}$ | - | 587.98 |
| octadec-9-ene | $C_{18}H_{36}$ | 583.15 | 587.98 |
| 7,11,15-trimethyl-3-methylene-hexadec-1-ene | $C_{20}H_{38}$ | 617.65 | 616.99 |
| pentacos-1-ene | $C_{25}H_{50}$ | 669.25 | 681.90 |

## CASE 2: SPARQL Query

The chemical class IRI representing "alkene" molecules can be linked to alkene species that miss the classification tag using the SPARQL update query in Figure S5. Alkene molecules are alkenes querying for species with sum formula $C_xH_{2x}$ that also have one double bond. The "FILTER NOT EXISTS" clause is used to checks if the species is linked to the instance of the "alkene" chemical class. If the link does not exist, then the solution will be included in the result set.

```
PREFIX os: <http://www.theworldavatar.com/ontology/ontospecies/OntoSpecies.owl#>
PREFIX rdf: <http://www.w3.org/1999/02/22-rdf-syntax-ns#>
PREFIX rdfs: <http://www.w3.org/2000/01/rdf-schema#>
PREFIX xsd: <http://www.w3.org/2001/XMLSchema#>

INSERT DATA {?speciesIRI os:hasChemicalClass ?classIRI}
WHERE {
   # select a species and with chemical formula CxH2x
   # ?formula - species chemical formula
   ?speciesIRI rdf:type os:Species ; rdfs:label ?formula .
   FILTER(regex(?formula, "C[0-9]{0,10}H[0-9]{0,10}$"))
   FILTER(xsd:float(strbefore(strafter(str(?formula),'C'),'H')) = 1/2*xsd:float(strafter(str(?formula),'H')))

   # select species that have a double bond
   ?speciesIRI os:hasAtomicBond ?bond .
   ?bond os:hasBondOrder 2 .

   # select species that miss the classification tag "alkene"
   FILTER NOT EXISTS{
      ?speciesIRI (rdf:|!rdf:)* ?x .
      ?x ?y ?z .
      ?z (rdf:|!rdf:)* ?classIRI .
      ?classIRI rdfs:label "alkene" .
   }
}
```

Figure S5: SPARQL query that find alkene species without classification and link to them the chemical class IRI that represents "alkene" molecules.

## CASE 4: SPARQL Query

Figure S6 shows a SPARQL query that identifies species with boiling point value entry that is 20% higher or lower than the recommended value.

```
PREFIX os: <http://www.theworldavatar.com/ontology/ontospecies/OntoSpecies.owl#>
PREFIX rdf: <http://www.w3.org/1999/02/22-rdf-syntax-ns#>
PREFIX rdfs: <http://www.w3.org/2000/01/rdf-schema#>

SELECT DISTINCT ?speciesIRI ?iupacstring
WHERE{
   ?speciesIRI rdf:type os:Species ; os:hasIUPACName ?iupac .
   ?iupac os:value ?iupacstring .
   FILTER(regex(str(?iupac) ,'_1_'))

   ?speciesIRI os:hasBoilingPoint ?BP1 .
   ?BP1 os:value ?BPvalue1 ;
      os:hasProvenance ?provenance .
   ?speciesIRI os:hasBoilingPoint ?BP2 .
   ?BP2 os:value ?BPvalue2 .
   ?provenance rdfs:label "PubChem agent" .
   FILTER( ?BPvalue2/?BPvalue1 > 1.2 || ?BPvalue2/?BPvalue1 < 0.8 )
}
```

Figure S6: SPARQL query selecting boiling point values of species that are 20% higher or lower than the recommended value.

## CASE 5: SPARQL Query

Figure S7 shows a federated SPARQL query that returns the HOMO-LUMO gaps and the level of theory and basis set of the DFT calculations performed for the carbon dioxide species.

```
PREFIX occ: <http://www.theworldavatar.com/ontology/ontocompchem/ontocompchem.owl#>
PREFIX os: <http://www.theworldavatar.com/ontology/ontospecies/OntoSpecies.owl#>
PREFIX rdf: <http://www.w3.org/1999/02/22-rdf-syntax-ns#>
PREFIX rdfs: <http://www.w3.org/2000/01/rdf-schema#>
PREFIX gc: <http://purl.org/gc/>

SELECT DISTINCT ?formula ?leveloftheory ?basisset ?homolumo
WHERE{
  # select CO2 species
  ?speciesIRI rdf:type os:Species ; rdfs:label ?formula .
  FILTER(str(?formula) = 'CO2')
  # retrive HOMO-LUMO info from OntoCompChem
  SERVICE <http://www.theworldavatar.com/blazegraph/namespace/ontocompchem/sparql> {
     ?calculation occ:hasUniqueSpecies ?speciesIRI ; gc:isCalculationOn ?lumo ; gc:isCalculationOn ?homo
     ↪  ; occ:hasInitialization ?i .
     ?i gc:hasParameter ?p1 ; gc:hasParameter ?p2 .
     ?p1 gc:hasBasisSet ?basisset .
     ?p2 occ:hasLevelOfTheory ?leveloftheory .
     ?lumo rdf:type occ:LumoEnergy ; occ:hasLumoEnergy ?lumoen .
     ?lumoen gc:hasValue ?lv .
     ?homo rdf:type occ:HomoEnergy ; occ:hasHomoEnergy ?homoen .
     ?homoen gc:hasValue ?hv .
     BIND( (xsd:float(?hv) - xsd:float(?lv))*27.2114 AS ?homolumo )
  }
}
```

Figure S7: Federated SPARQL query selecting the HOMO-LUMO gaps, and the level of theory and basis set of the DFT calculations performed to calculate those on the carbon dioxide species.

# References


(S1) McNaught, A.; Wilkinson, A. *IUPAC Compendium of Chemical Terminology*; Blackwell Scientific Publications: Oxford, UK, (the “gold book”) edition, 1997.

(S2) World Wide Web Consortium (W3C), Shapes Constraint Language (SHACL). 2023 (accessed April 25, 2023); `https://www.w3.org/TR/shacl/`.

(S3) Bakulin, I. K.; Orekhov, M. A. Basic Principles Underlying the Size Dependence of the Hydrocarbon Ionization Energy. *Journal of Experimental and Theoretical Physics* **2022**, *135*, 611–616.

(S4) Curzons, A.; Constable, D.; Cunningham, V. Solvent selection guide: a guide to the integration of environmental, health and safety criteria into the selection of solvents. *Clean Technologies and Environmental Policy* **1999**, *1*, 82–90.

(S5) Byrne, F. P.; Jin, S.; Paggiola, G.; Petchey, T. H. M.; Clark, J. H.; Farmer, T. J.; Hunt, A. J.; Robert McElroy, C.; Sherwood, J. Tools and techniques for solvent selection: green solvent selection guides. *Sustainable Chemical Processes* **2016**, *4*, 1–24.

(S6) Rihm, S. D.; Kovalev, M.; Lapkin, A. A.; Ager III, J. W.; Kraft, M. On the role of C4 and C5 products in electrochemical CO2 reduction via copper-based catalysts. *Energy & Environmental Science* **2023**, *16*, 1697–1710.
